# Two different mechanisms support selective attention at different phases of training

**DOI:** 10.1371/journal.pbio.2001724

**Published:** 2017-06-27

**Authors:** Sirawaj Itthipuripat, Kexin Cha, Anna Byers, John T. Serences

**Affiliations:** 1 Neurosciences Graduate Program, University of California, San Diego, La Jolla, California, United States of America; 2 Department of Psychology, University of California, San Diego, La Jolla, California, United States of America; 3 Kavli Institute for Brain and Mind, University of California, San Diego, La Jolla, California, United States of America; National Institute of Mental Health, United States of America

## Abstract

Selective attention supports the prioritized processing of relevant sensory information to facilitate goal-directed behavior. Studies in human subjects demonstrate that attentional gain of cortical responses can sufficiently account for attention-related improvements in behavior. On the other hand, studies using highly trained nonhuman primates suggest that reductions in neural noise can better explain attentional facilitation of behavior. Given the importance of selective information processing in nearly all domains of cognition, we sought to reconcile these competing accounts by testing the hypothesis that extensive behavioral training alters the neural mechanisms that support selective attention. We tested this hypothesis using electroencephalography (EEG) to measure stimulus-evoked visual responses from human subjects while they performed a selective spatial attention task over the course of ~1 month. Early in training, spatial attention led to an increase in the gain of stimulus-evoked visual responses. Gain was apparent within ~100 ms of stimulus onset, and a quantitative model based on signal detection theory (SDT) successfully linked the magnitude of this gain modulation to attention-related improvements in behavior. However, after extensive training, this early attentional gain was eliminated even though there were still substantial attention-related improvements in behavior. Accordingly, the SDT-based model required noise reduction to account for the link between the stimulus-evoked visual responses and attentional modulations of behavior. These findings suggest that training can lead to fundamental changes in the way attention alters the early cortical responses that support selective information processing. Moreover, these data facilitate the translation of results across different species and across experimental procedures that employ different behavioral training regimes.

## Author summary

Selective attention can enhance processing of sensory information via sensory gain and neural noise reduction. However, the extent to which these 2 mechanisms contribute to improvement in perceptual performance during attention is still debated. We hypothesized that the mechanisms that support selective attention depend on the duration of training for the task. Using electroencephalography (EEG), we measured over the course of 1 month visual responses from human subjects while they performed a selective spatial attention task and showed that attentional gain plays a dominant role early in training, but neural noise reduction dominates later in training. This observation has important implications for understanding attentional mechanisms as well as for generalizing results from studies using different model systems (e.g., human versus nonhuman primates) that often require substantially different amounts of training.

## Introduction

Selective attention influences sensory processing such that relevant information is preferentially encoded at the expense of irrelevant information. Over the last several decades, multiple electrophysiological and neuroimaging studies in humans and nonhuman primates have shown that attention selectively increases the amplitude, or the gain, of visual responses evoked by attended stimuli compared to responses evoked by unattended stimuli [1–35]. Many of these studies used electroencephalography (EEG) to measure population-level attentional gain in the human visual cortex by tracking changes in the amplitude of a visually-evoked event-related potential (ERP) waveform that peaks ~100 ms poststimulus, termed the P1 component [7,9,12,17–21, 31–32]. With respect to behavior and perception, attentional gain of the P1 component is correlated with improved target detection [19], with improved contrast discrimination thresholds [9], and with changes in perceived contrast [31], and taken together, these findings suggest that the amount of attentional gain in the human visual cortex has a significant impact on both perception and behavior during tasks that require selective attention.

In addition to the extensive literature on attention-related gain modulations, recent electrophysiological studies in nonhuman primates demonstrate that attention can also reduce the trial-by-trial variability of single neuron spike rates and the magnitude of correlated spiking between neurons [36–44]. Moreover, these modulations of neuronal noise may improve the signal-to-noise ratio of sensory codes more than attentional gain [37,40] and may be better predictors of improvements in behavioral performance [37]. While gain and noise modulations are not mutually exclusive, the degree to which each type of modulation impacts behavioral performance is difficult to evaluate given differences in experimental methods and training regimes used by different research groups [45–48]. For example, studies that focus on attentional gain as the dominant mechanism of selective attention typically use human participants who are trained for brief periods of time before data collection begins (typically less than 1 hour) [12,17–21, 31–32]. On the other hand, studies that focus on noise reduction typically use nonhuman primates who are trained for many months prior to recording neural activity [37,40,43]. Thus, training may play a key role in shaping how selective attention impacts cortical responses to facilitate goal-directed behavior, and characterizing training effects is also critical for generalizing results across tasks and species.

To directly test the influence of training on the neural mechanisms that support selective attention, we had human participants repeatedly perform a spatial attention task over the course of approximately 1 month (20 data recording sessions). Throughout training we used a standard psychophysical approach to measure behavioral performance, and we used EEG to measure stimulus-evoked responses over the visual cortex. Using human participants and EEG enabled us to immediately acquire data with relatively little initial practice so that we could monitor changes in both behavior and neural activity across training sessions. In addition, with EEG, we could measure changes in the amplitude of the P1 component as a marker of population-level gain modulations in extrastriate regions of the visual cortex [7,9,12,17–21, 31–32]. We focused on the P1 component because it is sensitive to changes in stimulus intensity and to attentional manipulations [7,9,12,17–21, 31–32]. Moreover, recent studies have demonstrated that sensory and attentional modulations of spiking activity in the extrastriate visual cortex are correlated with modulations of population-based measures such as the local field potential (LFP) in the same temporal window as the P1 in both nonhuman primate and human subjects [49–50]. Given that the P1 is thought to be closely correlated with LFP modulations, the present approach thus provides access to both behavioral and cortical responses across a wide range of training phases.

Consistent with prior results in human subjects using EEG and spatial attention tasks, early in training we observed attentional facilitation of behavioral performance and a robust attentional gain modulation of the P1 component [7,9,12,17–21,31–32]. However, later in training, there was still an attentional facilitation of behavioral performance even though attentional gain of the P1 component vanished. Next, we adopted a standard quantitative model, based on signal detection theory (SDT), to infer whether attentional gain or noise reduction best predicted the relationship between attentional modulations of the P1 component and behavior across training sessions [5,9,28,47,51–53]. Over the course of training, the SDT model suggests a link between P1 gain and behavioral gain early in training but noise reduction later in training. This putative transformation from attentional gain to noise reduction suggests that different neural mechanisms may support selective attention at different phases of training.

## Results

### Behavioral results

**Fig 1:**
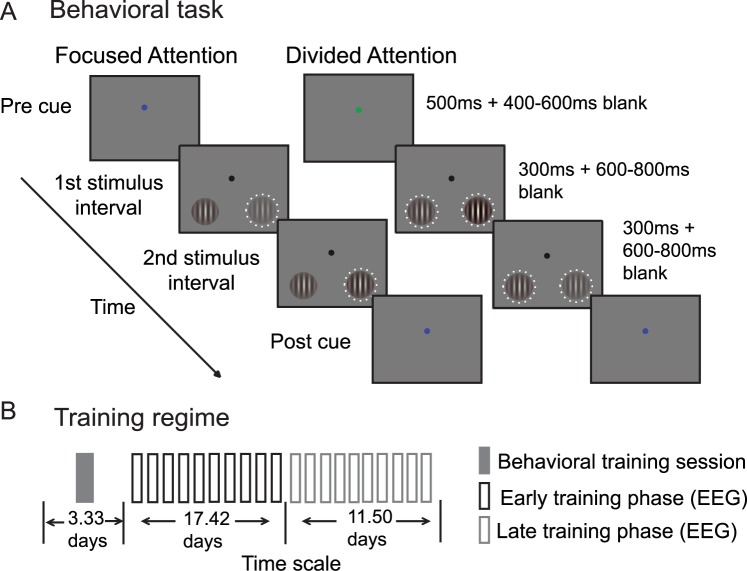
Task design. (A) A 2-interval force choice (2IFC) contrast discrimination task that required either focused or divided spatial attention. (B) Training regime: 12 participants completed 1 session of behavioral training and then completed 20 sessions of simultaneous behavioral testing and electroencephalography (EEG) recording over an average of 32.25 days. For the main analysis, we defined the first 10 EEG recording sessions as the “early phase” of training and the last 10 sessions as the “late phase” of training.

Human participants performed a 2-interval forced choice (2IFC) contrast discrimination task (Fig 1A). We used this task to make contact with previous studies in both humans and nonhuman primates that have employed similar paradigms [5,9,36–38]. At the start of a trial, subjects were cued to attend to either the left or right lower visual quadrant (termed focused attention trials), or they were cued to attend to both locations (termed divided attention trials). The cue was followed by 2 successive stimulus intervals, and each interval contained 1 sinusoidal Gabor stimulus to the left and 1 to the right of fixation. In 1 of the 2 stimulus-presentation intervals, the contrast of each stimulus was pseudorandomly drawn from 0%–61.66% Michelson contrast. We refer these contrast values as “pedestal” contrast values. In the other stimulus interval, we added a slight contrast increment to the pedestal contrast value of 1 of the 2 stimuli, and participants then had to report whether the first or the second interval contained the stimulus with a higher contrast. In the focused attention condition, the 2 successive stimuli at the cued location were always rendered at different contrast values. At the uncued location, the 2 successive stimuli were always rendered at the same contrast. We refer to stimuli presented in the cued location as “focused target” stimuli and stimuli presented in the uncued location as “focused nontarget” stimuli. In the divided attention condition, both locations were equally likely to contain the contrast change, yielding “divided target” and “divided nontarget” stimuli. Importantly, the pedestal contrast value presented in the target location was paired an equal number of times with nontarget stimuli rendered at each possible contrast value. The main dependent measure was the change in contrast (i.e., contrast threshold or Δ*c*) from each pedestal contrast that was required to achieve an accuracy level of 76% (perceptual sensitivity or d’ of approximately 1). When paired with simultaneous EEG recording, this psychophysical approach allowed us to derive both psychometric and neurometric response functions so that we could directly link attentional modulations in neural responses with attentional modulations in behavior (see also [5,9,28,47,51–53]).

**Fig 2:**
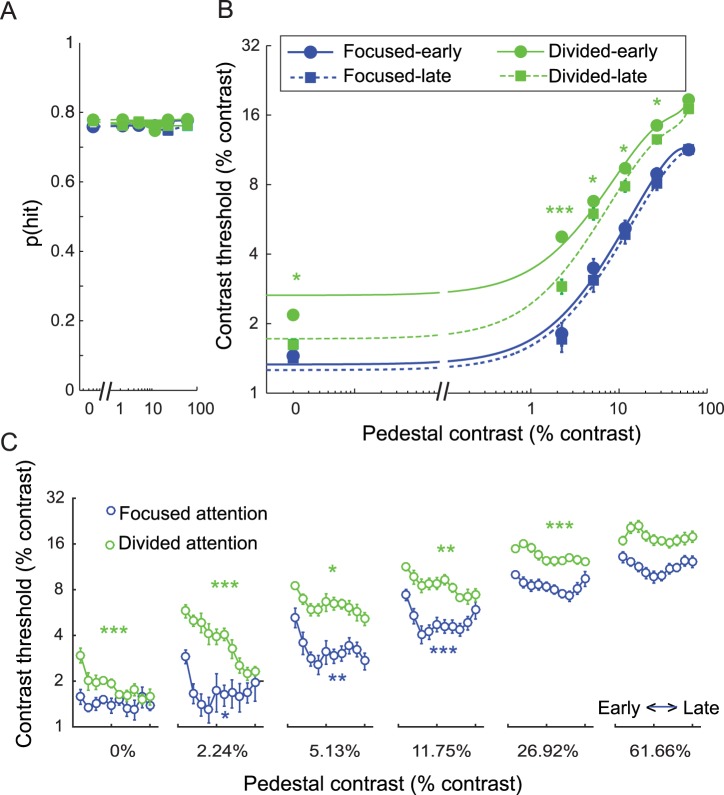
Behavioral results. (A) The hit rate was fixed at ~76% (d’ = ~1) across all conditions so that contrast discrimination thresholds could be measured as a function of attention and training. Error bars represent within-subject SEM. (B) Psychophysical contrast discrimination thresholds (Δc) for stimuli across all pedestal contrast levels in the focused and divided attention conditions split into early and a late training phases (10 electroencephalography [EEG] sessions for each phase). Error bars represent within-subject SEM. Green *, **, and *** in (B) represent pairwise differences between contrast thresholds in the divided attention condition across training phases with *p*’s < 0.05, < 0.01, and < 0.001, respectively (false discovery rate [FDR]-corrected). (C) The day-by-day Δc data showing a faster learning rate in the focused attention condition compared to the divided attention condition (2 EEG sessions or ~1 day of experiment per time bin). Error bars represent within-subject SEM. Blue and green *, **, and *** in (C) represent significant changes in Δc across days in the focused and divided attention conditions with *p*’s < 0.05, < 0.01, and < 0.001, respectively (FDR-corrected). Data are available from the Open Science Framework (https://osf.io/pc7dr/).

To evaluate the effects of training on attentional modulations of cortical responses, 12 human participants participated in 20 EEG sessions over the course of approximately 1 month (Fig 1B, see Materials and methods, subsection Subjects). Note that on most days, most subjects participated in 2 EEG sessions (10 subjects completed 2 sessions every day, 1 subject completed 2 sessions most days except for 1 session for 1 day and 3 sessions for another day, and the other subject completed 2 sessions most days except for 1 session for 3 days and 3 sessions for another day). In each experimental session, we estimated the incremental contrast value (Δ*c*) required to reach criterion performance at each pedestal contrast and attention condition (Fig 2A; mean hit rate: 76.6% ± SEM 0.3%, yielding d’ = ~1).

Fig 2B illustrated the psychophysical contrast thresholds (Δc) required to achieve a fixed hit rate (Fig 2A) as a function of pedestal contrast in the focused attention and divided attention conditions (to produce a threshold versus contrast [TvC] curve). The behavioral data presented here were divided into 2 training phases in order to match the data from the main EEG analysis, in which we tried to obtain ~400 trials for each experimental condition (10 EEG sessions for each training phase) as suggested by Luck [54]. In line with previous studies, Δc increased as a function of contrast (F(5, 55) = 143.38, *p* < 0.001) [9,28,52,55–58] and decreased with focused compared to divided attention (F(1, 11) = 137.95, *p* < 0.001) [5,9,28,56]. These attentional modulations occurred at all contrast levels (all t(11)’s ≥ 5.03, all *p*’s < 0.001, data collapsed across early and late training phases, false discovery rate [FDR]-corrected, 2-tailed). In addition, there were significant attention effects on Δc in both training phases (t(11)’s = 11.23 and 9.28 for early and late training phases, respectively, collapsed across contrasts, all *p*’s < 0.001). Finally, training also decreased Δc (F(1, 11) = 9.85, *p* = 0.009). This training effect on Δc was driven primarily by improved performance in the divided attention condition (F(1, 11) = 11.96, *p* = 0.005) for the stimuli of 0%–26.92% contrast levels (t(11)’s = 2.07–4.70; *p*’s ≤ 0.031, FDR-corrected, 1-tailed under an assumption that training should improve behavioral performance).

To examine attention and training effects on a finer timescale, we sorted the behavioral contrast discrimination threshold data into 10 time bins (2 EEG sessions or ~1 day for each bin). Consistent with the main result, there were significant main effects of attention across all phases of training (Fig 2C). Paired *t* tests comparing data from the focused attention and divided attention conditions, collapsed across all contrast levels, revealed significant mean differences in behavioral contrast thresholds across all time bins (t(11)’s = 5.05–14.26, *p*’s < 0.001, FDR-corrected, 1-tailed under the assumption that focused attention should lead to improved behavioral performance compared to divided attention). Moreover, we also found that there were learning effects not only in the divided attention condition but also in the focused attention condition. However, these learning effects occurred at a much faster rate than in the divided attention condition. To evaluate these learning effects, we performed separate 1-way, repeated-measures ANOVAs with day as a within-subject factor on data from each contrast level in the divided attention and the focused attention conditions (12 tests in total, FDR-corrected across all tests). In the divided attention condition, we found significant training effects for pedestal contrasts ranging from 0%–26.92% (F(9,99)’s = 2.47–7.61 with all *p*’s ≤ 0.014) but not for a pedestal contrast of 61.66% (F(9,99) = 1.28 with *p* = 0.259). In the focused attention condition, we found significant training effects for pedestal contrasts ranging from 2.24%–11.75% (F(9,99)’s = 2.43–4.71 with *p*’s ≤ 0.015) but not for the other contrast levels (F(9,99)’s = 0.49–1.92 with *p*’s = 0.058–0.877). The impact of training on behavior in the focused attention condition primarily occurred in between the first day and the second day and was thus earlier and more abrupt than training effects in the divided attention condition. Post hoc pairwise *t* tests revealed a significant effect between the first and the second days for pedestal contrasts of 2.24%–11.75% (t(11)’s = 3.82 to 4.52, *p*’s ≤ 0.001, FDR-corrected, 1-tailed under the assumption that training should improve behavioral performance). After the second day, behavioral performance stabilized for the duration of the experimental sessions and post hoc pairwise *t* tests revealed no significant differences between the second day and any of the following days for pedestal contrasts of 2.24%–11.75% (t(11)’s = –0.58 to 2.45 with *p*’s = 0.016 to 0.483, nonsignificant after FDR correction, 1-tailed under the same assumption). Note that we only performed post hoc *t* tests on the data associated with 2.24%–11.75% pedestal contrasts given that only these conditions exhibited significant training effects, as evaluated earlier using 1-way ANOVAs.

### EEG results: General analysis approach

**Fig 3:**
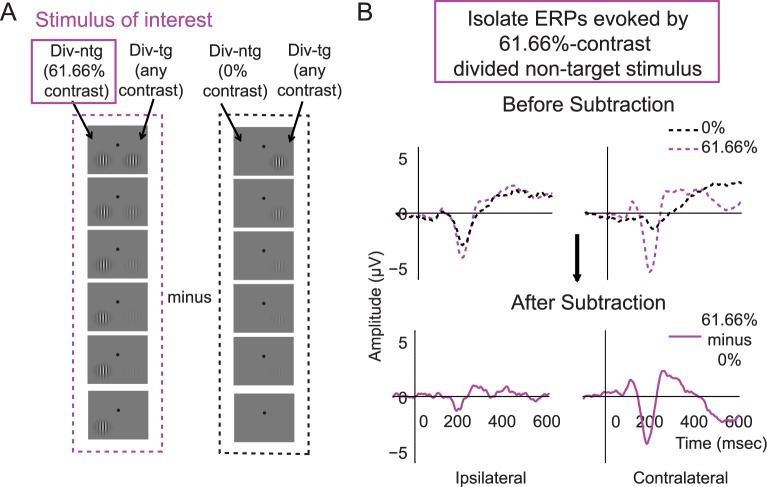
An example of the event-related potential (ERP) subtraction method. (A) Left column (in purple): schematic of a 61.66% contrast divided nontarget stimulus presented in the left hemifield (termed the stimulus of interest) and paired with target stimuli rendered in all different contrasts (down the rows). Right column (in black): the divided nontarget 0% contrast stimulus in the left hemifield paired with the same set of target stimuli in the right hemifield. (B) In this case, the ERP response evoked by the left divided nontarget stimulus of 0% contrast (A, right; B, top, black dotted traces) was subtracted from the ERP response evoked by the left divided nontarget stimulus of 61.66% contrast (A, left; B, top, dotted purple traces), resulting in the baseline-subtracted ERP response (B, bottom, solid purple traces). A similar subtraction was done to compute the ERPs associated with stimuli of interest rendered at all other contrasts. Note that the stimulus paired with the stimulus of interest (in this case, the right divided target stimulus) was equally likely to have any of the 6 contrast values. Therefore, this method amounts to subtracting out the mean response to all ipsilateral stimuli.

**Fig 4:**
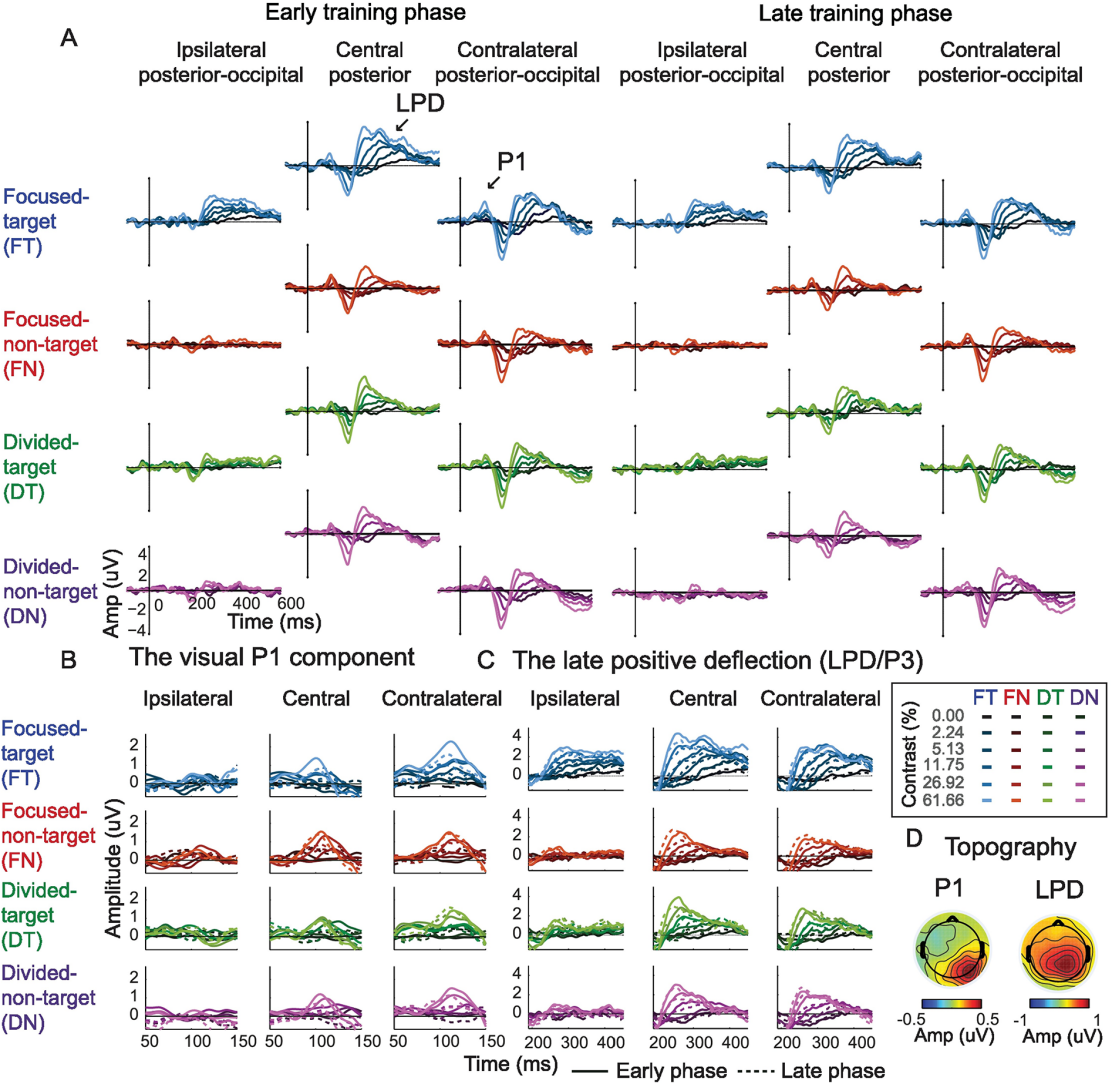
Event-related potential (ERP) results. (A) ERP traces evoked by the focused target, focused nontarget, divided target, and divided nontarget conditions across early and late training phases. The shading of the colors represents the contrast level of the stimulus (dark to bright colors represent low to high contrast levels). The ERP subtraction method, which helped isolate the ERPs evoked by the stimulus of interest, is illustrated in Fig 3. (B) Zoom-in figure of the visual P1 component. (C) Zoom-in figure of the late positive deflection (LPD or P3). (D) Topographical maps of the P1 and the LPD component collapsed across all experimental conditions. The left and the right sides of the topographical map depict the response in electrodes that are ipsilateral and contralateral to the stimulus of interest, respectively. Data are available from the Open Science Framework (https://osf.io/pc7dr/).

To examine cortical responses evoked by attended and unattended stimuli, we focused on quantifying the P1 response averaged across the first and second stimulus presentation intervals (and note that we also examined the first and second intervals separately, see results below). The early visual system has a contralateral mapping between external stimuli and their cortical representation, such that stimuli presented in the left visual field evoke responses in right occipital cortex and vice versa. However, EEG has relatively coarse spatial resolution, thus ERPs recorded over the occipital lobe typically reflect a mixture of responses evoked by both stimuli (unless a 0% contrast stimulus was presented in 1 hemifield). Thus, to better isolate the stimulus-evoked responses associated with stimuli presented on the left and right sides of space, we first averaged ERPs on trials that had a 0% contrast stimulus in the visual field contralateral to a given electrode of interest and a nonzero contrast stimulus in the ipsilateral visual field. The resulting averaged ERP thus reflects the average degree of “spillover” from stimuli of all possible contrast levels, which were presented in the ipsilateral visual field. Next, we subtracted this ERP from the ERPs on trials in which nonzero contrast visual stimuli were presented in the contralateral visual field (Fig 3) (see similar methods in [9,31,59]). This subtraction method was performed separately for each contralateral stimulus contrast level, each attention condition, and each training phase. This method helped not only to isolate evoked responses associated with a single stimulus from a bilateral stimulus array but also to control for any spatially nonspecific anticipatory effects associated with the presentation of attention cues [9,31,59]. As shown in Fig 4 and S1 Fig, the P1 components peaked in the contralateral posterior–occipital electrodes ~80–130 ms poststimulus, consistent with previous ERP studies [7,9,12,17–21, 31–32]. Note that the validity of this baseline subtraction method relies on an assumption of linearity, which appears to be reasonable in this situation. First, we counterbalanced the contrasts of target and nontarget stimuli so that there were equal numbers of trials in which each possible target contrast value was paired with each possible nontarget contrast value (and vice versa). Therefore, this method amounts to subtracting out the average response to ipsilateral stimuli across all contrast values. Next, to further ensure that the pattern of results was not artificially influenced by the subtraction method, we also analyzed the data without baseline subtraction and we observed the similar pattern of results, thus validating the linearity assumption of the baseline subtraction method (see below).

### Training attenuates attentional gain of the visual P1 component

**Fig 5:**
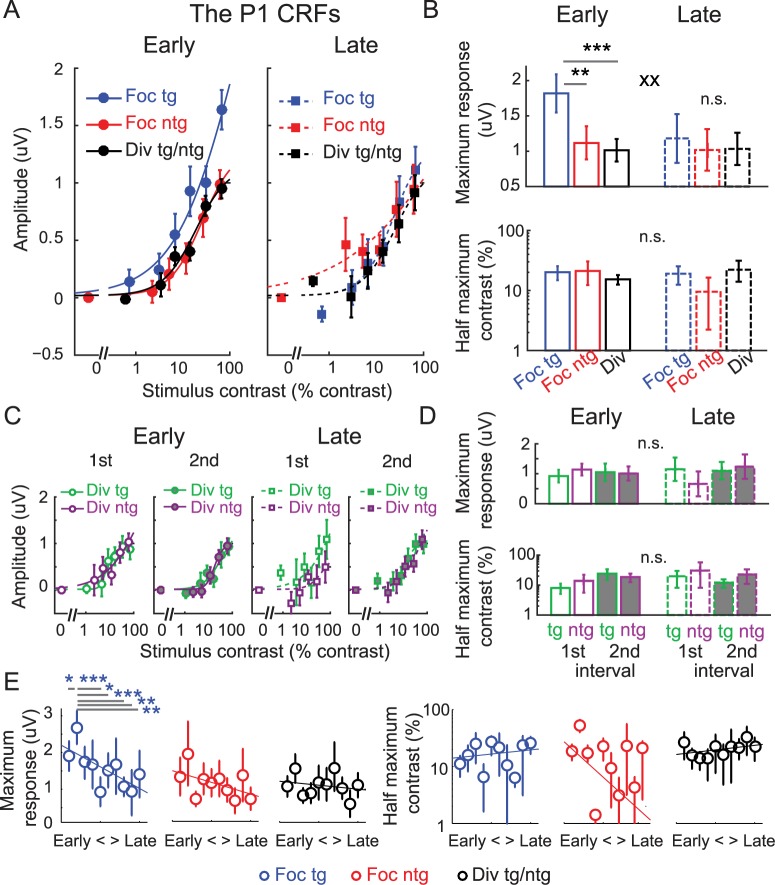
Modulations of the P1 component with baseline subtraction. The contrast response functions (CRFs), based on the amplitude of the extracted P1 component, which was averaged over the contralateral posterior–occipital electrodes from 80–130 ms poststimulus. During the early training phase, there was a robust attentional gain amplification of the P1 component on focused attention trials compared to divided attention trials (left panel). However, no attention-related gain modulations were present during the late training phase (right panel). Error bars represent within-subject SEM. (B) Corresponding maximum response (response at 100% contrast minus baseline) and half-maximum contrast for the P1-based CRFs in (A). Error bars represent the 68% CIs; ** and *** represent significant pairwise comparisons between attention conditions in each training phase with *p* < 0.01 and *p* < 0.001, respectively. Note that in this and subsequent figures, we use 68% CIs for data that were analyzed via nonparametric bootstrapping methods (e.g., the fit parameters) and within-subject SEM for data that were analyzed using standard parametric approaches. ^xx^ represents a significant interaction between training (early and late) and attention (focused target and divided attention), with *p* < 0.01. (C) The P1-based CRFs evoked by the divided target and divided nontarget stimuli during the first and second stimulus intervals across the early and late training phases. There was no significant change in the P1-based CRFs across these stimulus conditions and training phases. This justified averaging between the P1 data elicited by the divided target and divided nontarget stimuli from both stimulus intervals. Error bars represent within-subject SEM. (D) Corresponding maximum response and half-maximum contrast for the P1-based CRFs in (C). Error bars represent the 68% CIs. (E) The day-by-day analysis of the P1 data with baseline subtraction (2 electroencephalography [EEG] sessions or ~1 day per each time bin). Left and right panels represent the estimated maximum response and half-maximum contrast parameters. Error bars represent the 68% CIs. Blue *, **, and *** in (E) represent significant pairwise comparisons between the maximum response on the second time bin in which the behavioral performance started to reach a saturation level (Fig 2C) and the maximum response in other time bins with *p* < 0.05, *p* < 0.01, and *p* < 0.001 (false discovery rate [FDR]-corrected). Note that the contrast values on the x-axis in (A) and (C) are not exactly the same across target and nontarget conditions because in the target conditions, we used the averaged contrast values of the pedestal and incremental stimuli. Data are available from the Open Science Framework (https://osf.io/pc7dr/). n.s., not significant.

Fig 5A shows the mean P1 amplitude from 80–130 ms poststimulus over the contralateral posterior–occipital electrodes as a function of stimulus contrast, yielding contrast response functions (CRFs) for focused target, focused nontarget (or ignored stimulus), and divided attention conditions (averaged across the divided target and divided nontarget conditions). We then characterized the shape of the CRF in each condition using a Naka–Rushton equation (see Eq 1 and Materials and methods) to estimate the maximum response (response at 100% contrast minus baseline) and the half-maximum contrast value that determines the horizontal position of the CRF along the x-axis (Fig 5B). To examine changes in the CRF fit parameters, we used resampling statistics (see details in Materials and methods). Note that averaging data across the divided target and divided nontarget conditions was justified based on the fact that there was no main effect of stimulus type, no main effect of stimulus interval, no main effect of training, or no interaction between these factors on the maximum response and the half-maximum contrast of the P1-based CRFs (all *p*’s ≥ 0.248, resampling tests, 2-tailed; see Materials and methods) (Fig 5C and 5D).

Consistent with a recent report [9], early in training we found a significant increase in the maximum response of the P1-based CRFs in the focused target condition compared to the focused nontarget condition (*p* = 0.004) and a significant increase in the maximum response in the focused target condition compared to the divided attention condition (Fig 5A left and 5B top, *p* < 0.001, resampling tests, 2-tailed). In contrast, later in training we found no difference in the maximum response between the focused target and the focused nontarget conditions (*p* = 0.694) or between the focused target and the divided attention conditions (Fig 5A right and 5B top, *p* = 0.687, resampling tests, 2-tailed). This overall pattern gave rise to a significant interaction between training (early and late) and attention conditions (focused target and divided attention) (*p* = 0.006, resampling test, 1-tailed because of the assumed direction of the interaction). Importantly, we found that the attenuated attentional gain after training was due to a selective reduction of the maximum P1 response in the focused target condition (*p* = 0.013), accompanied by no changes in either the focused nontarget condition (*p* = 0.795) or the divided attention condition (*p* = 0.935, resampling tests, 2-tailed). The fact that the training-related change in P1 amplitude was specific to the focused target condition suggests that training specifically impacted neural modulations related to the deployment of focused attention. Moreover, the specificity of these modulations indicates that training-related changes in P1 amplitude were not due to general low-level sensory or perceptual learning effects since training did not impact the magnitude of the P1 associated with any other condition (i.e., the focused nontarget and divided attention conditions).

We next performed a follow-up EEG analysis to more precisely characterize the timecourse of training effects by sorting the EEG data into 10 time bins (2 EEG sessions per bin or ~1 day per bin). Fig 5E shows the results of this analysis, which support the same conclusions as the split-half data broken down into early and late training phases. In the day-by-day data, training led to a significant reduction in the maximum response of the P1-based CRFs in the focused-target condition (i.e., the slope of the linear fit across 10 time bins was significantly less than 0, a resampling test with *p* = 0.004, 2-tailed, see Materials and methods), while the maximum response of the focused nontarget and divided attention CRFs did not change with training (resampling tests with *p*’s = 0.348 and 0.427, respectively, 2-tailed). Interestingly, the significant effect of training in the focused target condition was driven by a marked increase in the maximum CRF response on the second day, the same day that behavioral performance in the focused attention condition dramatically improved (Fig 2C). On the third day, when behavioral performance started to stabilize in the focused attention condition, there was a corresponding drop in the maximum P1 response. Post hoc pairwise comparisons confirmed that the maximum response on the second day was significantly higher than the maximum response in the first day (*p* = 0.030) and higher than the maximum response values on the fifth, sixth eighth, ninth, and 10th days (all *p*’s ≤ 0.029, resampling tests, FDR-corrected, 1-tailed; see Materials and methods). This pattern of rising and falling early visual responses that occurred in parallel with the improvement and stabilization of perceptual performance is consistent with findings previously reported by a study on perceptual learning [60] (see Discussion).

While training had a significant impact on the attentional modulation of the maximum response of the P1-based CRFs, we did not find changes in the half-maximum contrast across any comparison between the focused target, focused nontarget, and divided attention conditions during the early or late training phase (Fig 5B, bottom, all *p*’s ≥ 0.156; resampling tests,2-tailed). In addition, we did not find any training-related changes in the half-maximum contrast of the P1-based CRFs in the focused target (*p* = 0.893), focused nontarget (*p* = 0.412), or divided attention condition (*p* = 0.333, resampling tests, 2-tailed). The follow-up day-by-day EEG analysis also revealed no changes in the half-maximum contrast across 10 time bins: the slopes of the linear fit across 10 time bins were not significantly different than 0 (*p*’s = 0.655, 0.255, and 0.535 for the focused target, focused nontarget, and divided attention conditions, respectively, resampling tests, 2-tailed). Collectively, these results suggest that training primarily impacts the degree to which attention amplifies the maximum response of population-level stimulus-evoked responses.

**Fig 6:**
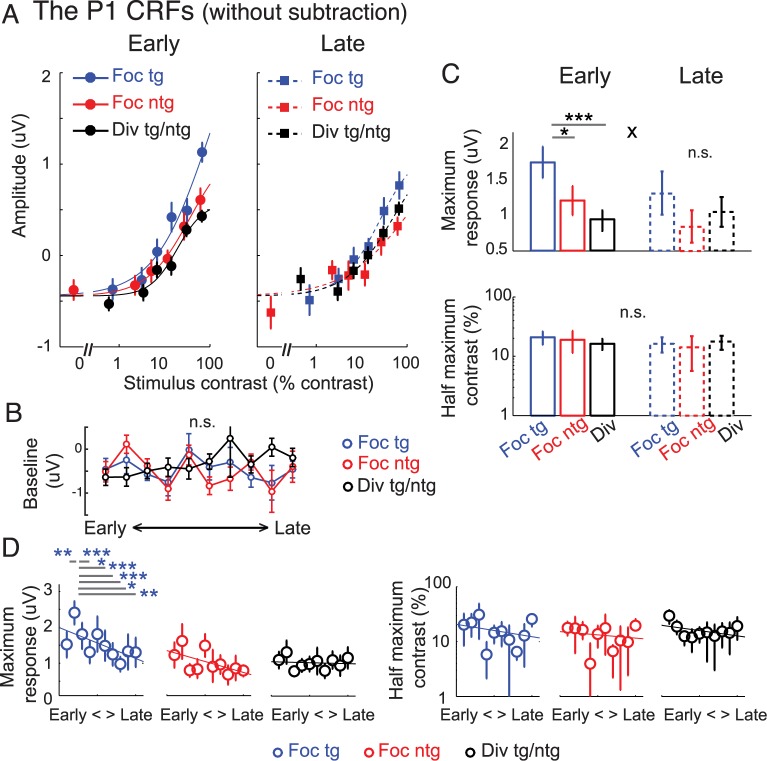
Modulations of the P1 component without baseline subtraction. (A) The P1 contrast response functions (CRFs) based on event-related potentials (ERPs) without the baseline subtraction. Note that the contrast values on the x-axis are not exactly the same across target and nontarget conditions because in the target conditions we used the averaged contrast values between the pedestal and incremental stimuli. (B) The P1 response amplitude obtained from 0% pedestal contrast stimuli (i.e., baseline activity) across training days (2 electroencephalography [EEG] sessions or ~1 day per time bin). Error bars in (A) and (B) represent within-subject SEM. There was no main effect of attention, no main effect of training, or no interaction between the 2 factors on the P1 baseline activity. (C) Corresponding maximum response (response at 100% contrast minus baseline) and half-maximum contrast for the P1-based CRFs in (A). Overall, the results are consistent with the P1 results with baseline subtraction (Fig 5), in which a gain modulation of the maximum response was observed during the early training phase, but this attentional gain modulation disappeared after training. The main difference between this result and the baseline-subtracted result is that the baselines of the P1 CRFs across all experimental conditions were shifted down and were negative. This was due to an early negative component induced by the stimulus that was ipsilateral to the electrode of interest. Error bars represent the 68% CIs; * and *** represent significant pairwise comparisons between attention conditions in each training phase with *p* < 0.05 and *p* < 0.001, respectively. ^x^ represents a significant interaction between training (early and late) and attention (focused target and divided attention) with *p* < 0.05. (D) The day-by-day analysis of the P1 data without baseline subtraction (2 EEG sessions or ~1 day per each time bin). Left and right panels represent the estimated maximum response and half-maximum contrast parameters. Error bars represent the 68% CIs. Blue *, **, and *** in (D) represent significant pairwise comparisons between the maximum response on the second day, on which the behavioral performance started to reach a saturation level (Fig 2C), and the maximum response on other time bins, with *p* < 0.05, *p* < 0.01, and *p* < 0.001 (false discovery rate [FDR]-corrected). Data are available from the Open Science Framework (https://osf.io/pc7dr/). n.s., not significant.

For comparison, we also analyzed the P1-based CRFs without subtracting the baseline activity. As illustrated in Fig 6, the results were qualitatively similar to the results obtained using the subtraction method (Fig 5). Thus, this validated the linearity assumption of the baseline subtraction method. First, we fit the Naka–Rushton equation (Eq 1) to characterize the CRFs, but this time we included an additional free parameter to account for baseline differences between conditions. We then performed a nested model comparison to assess the goodness of fit between the model that allowed baseline parameters to change freely and the model that fixed the baseline parameter across all experimental conditions (see Materials and methods). This analysis revealed that allowing baseline parameters to change freely did not significantly improve the goodness of fit (F(5, 11) = 0.97, *p* = 0.479, nested test). This suggests that baseline parameters associated with the P1-based CRFs did not change with attention or with training. Further supporting this result, a day-by-day EEG analysis comparing the P1 response amplitude obtained from trials of 0% pedestal contrast across 10 time bins (2 EEG sessions or ~1 day each bin) also suggests that baseline activity did not change with attention or training (Fig 6B). Specifically, a 2-way repeated-measures ANOVA showed no main effect of training (10 time bins: F(9, 99) = 1.00, *p* = 0.447), no main effect of attention (focused target, focused nontarget, and divided attention conditions: F(2, 22) = 0.71, *p* = 0.501), and no interaction between these 2 factors on the P1 amplitude in trials with stimuli of 0% pedestal contrast (i.e., baseline activity) (F(18, 198) = 0.90, *p* = 0.574). The null attention and training effects on the P1 baseline activity speaks against the possibility that attention and training might induce changes in perceptual template in the early visual cortex, which may have caused subjects to see stimuli when none were presented.

Consistent with the P1 data that was subjected to the baseline subtraction procedure (Fig 5), we observed the same pattern of response modulation with attention during the early training phase that dissipated after extended training. Thus, this further verified the linearity assumption of the baseline subtraction method (discussed in the EEG results: General analysis approach section). As illustrated in Fig 6C (top), there was a significant increase in the maximum response of the P1-based CRFs for the focused target condition compared to the focused nontarget condition (*p* = 0.018) and a significant increase in the maximum response in the focused target condition compared to the divided attention condition (*p* < 0.001, resampling tests, 2-tailed). In contrast, later in training we found no difference in the maximum response between the focused target and the focused nontarget conditions (*p* = 0.113) or between the focused target and the divided attention conditions (*p* = 0.367, resampling tests, 2-tailed). This training-induced reduction in the attentional modulation of the maximum P1 response gave rise to a significant interaction between the training and attention conditions (*p* = 0.034, resampling test, 1-tailed because of the assumed direction of the interaction). A follow-up analysis of the day-by-day EEG data also revealed a trend toward decreasing amplitude of the maximum response associated the P1 CRFs in the focused target condition across training sessions (*p* = 0.063, 2-tailed). In contrast, the maximum responses of the CRFs associated with focused nontarget (*p* = 0.107) and divided attention conditions remained unchanged (*p* = 0.730, resampling tests, 2-tailed). Similar to the results with baseline subtraction, we also found that the maximum response of the P1-based CRFs in the focused target condition was the highest on the second day, and it was significantly higher than the maximum response values on the first day (*p* = 0.010) and later days, including the fourth, sixth, seventh, eighth, ninth, and 10th days (all *p*’s ≤ 0.023, resampling tests, FDR-corrected, 1-tailed; see Materials and methods).

In addition, the half-maximum contrast associated with the P1 without baseline subtraction was unchanged across attention conditions and training phases (all *p*’s ≥ 0.425, resampling tests, 2-tailed). Also, the day-by-day EEG analysis revealed no changes in the half-maximum contrast across 10 time bins: the slopes of the linear fits across 10 time bins were not significantly different than 0 (*p*’s = 0.600, 0.841, and 0.351 for the focused target, focused nontarget, and divided attention conditions, respectively).

### Quantitative modeling suggests a transition from gain to noise reduction after training

Overall, the P1 results suggest that attentional gain is a prominent mechanism that supports attentional selection early in training. However, the absence of attentional gain later in training suggests that training may alter the neural mechanisms that support attentional selection. For example, recent studies propose that noise reduction or efficient decoding schemes can facilitate selective processing even in the absence of gain [5,28,36–37,40,43–44,51]. To evaluate these alternative accounts, we adopted a quantitative modeling framework based on SDT to evaluate the impact of gain and noise modulations on behavioral performance [5,9,28,47,51–53]. Later, we also evaluated possible contributions of efficient decoding [5,28].

**Fig 7:**
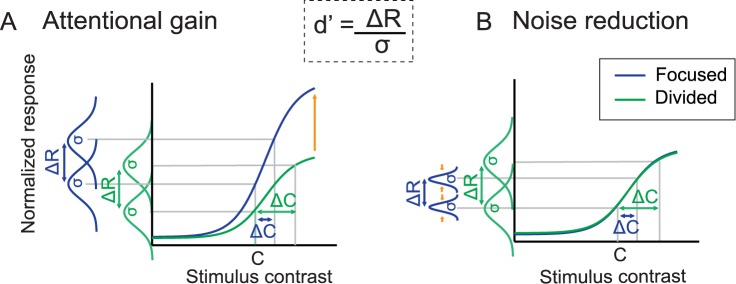
Competing attention mechanisms based on signal detection theory (SDT). According to SDT, perceptual sensitivity (d’) increases via increasing gain (ΔR) and via reducing noise (σ). (A) Attentional gain models posit that selective attention enhances the magnitude of early visual responses (ΔR), which leads to an increase in perceptual sensitivity (d’). (B) Noise reduction models hold that attention reduces trial-by-trial neuronal variability and improves d’ by reducing σ. We hypothesized that training might qualitatively alter the neural mechanisms that support attentional selection, with a shift from attentional gain (A) to noise reduction (B) over time.

As illustrated in Fig 7 and Eq 3, The SDT-based model posits that perceptual sensitivity (or d’) is determined by the difference between the mean neural responses evoked by 2 different stimuli (ΔR) divided by the trial-by-trial variability of those responses (σ) [5,9,28,47,51–53]. Attentional gain can increase d’ by increasing ΔR (Fig 7, left), and noise reductions can increase d’ by decreasing σ (Fig 7 right). Thus, this framework allowed us to make inferences about the importance of gain and noise modulations even though direct measures for neuronal noise are not available because of the noninvasive nature of EEG. We then used this model to formally link observed contrast discrimination thresholds with observed modulations of the P1 response. For example, when d’ is fixed and there is a decrease in psychophysical contrast thresholds (Δc) with attention, a model based solely on changes in attentional gain predicts an increase in the maximum response of the neural CRFs in the focused target compared to the divided attention conditions. In turn, if the predicted neural gain changes are too large or too small compared to the observed data, then the model can incorporate changes in σ to improve the link between neural CRFs and behavior.

**Fig 8:**
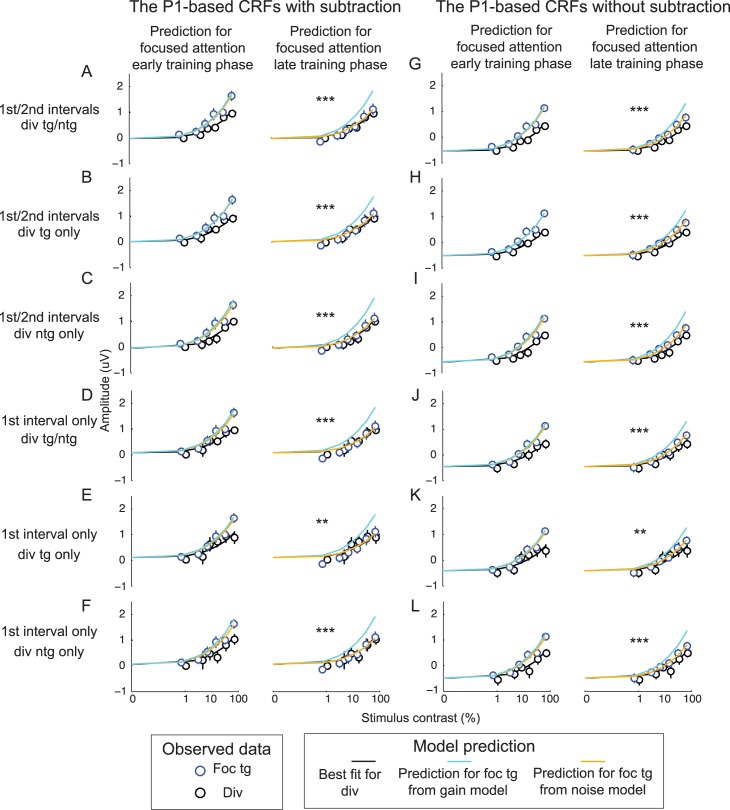
Linking changes in the psychophysical data and the P1 data using gain and noise models. A quantitative model based on signal detection theory (SDT) revealed that, early in training, attention-induced improvements in behavioral performance were sufficiently explained by the gain model, and the noise model did not significantly improve the fit (left panels in all subfigures). However, later in training, the noise model provides a significantly better prediction than the gain model (right panels in all subfigures). The modeling results are consistent across all modeling routines in which the P1-based contrast response functions (CRFs) in the divided attention condition were obtained from the average between the divided target and divided nontarget conditions (A, D, G, and J), from the divided target condition only (B, E, H, and K), from the divided nontarget condition only (C, F, I, and L), from the average between 2 stimulus intervals (A–C and G–I), and from the first stimulus interval only (D–F and J–L). Last, (A–F) show data with the baseline subtraction and (G–L) show data without baseline subtraction. ** and *** represent significant improvements with the noise reduction model p < 0.01 and p < 0.001 (false discovery rate [FDR]-corrected). See corresponding model parameters in Tables 1 and 2. Data are available from the Open Science Framework (https://osf.io/pc7dr/).

Consistent with a recent study [9], the gain model effectively linked changes in contrast thresholds and changes in the slope of the P1-based CRFs during the early training phase (compare a cyan curve and blue circles in the left panel of Fig 8A and see Table 1 for corresponding fitting parameters). Moreover, the noise model did not significantly improve the fit between the behavioral data and the P1-based CRFs compared to the gain model (compare orange and cyan curves; F(1, 8) = 1.12, *p* = 0.322, nested test). This suggests that attentional gain sufficiently accounts for the relationship between attentional modulations in neural and behavioral data early in training. However, later in training there was no attentional modulation of P1 amplitude (the right panel of Fig 8A) even though there was still an improvement in behavior with focused attention (Fig 2B). Thus, the gain model overestimated the slope of the P1-based CRFs in the focused target condition during the late training phase (compare the cyan curve and blue circles in the right panel of Fig 8A and see Table 2 for corresponding fit parameters). Instead, the noise model provided a significantly better fit compared to the gain model (compare orange and cyan curves; F(1, 8) = 89.18, *p* < 0.001, nested test). Importantly, we observed consistent results when we used the P1 data averaged across the divided target and divided nontarget conditions (Fig 8A and 8D), the P1 data from the divided target condition only (Fig 8B and 8E), the P1 data from the divided nontarget condition only (Fig 8C and 8F), and the P1 data from the divided target condition from just the first stimulus interval (Fig 8A–8F). Moreover, we obtained similar results using P1 data without baseline subtraction (Fig 8G–8L and Tables 1 and 2).

**Table 1:** Corresponding modeling results during the early training phase in Fig 8.

Figure		Early training phase	
	Gain model	Noise model	Nested model
	R^2^/baseline/noise	R^2^/baseline/noise	F value/*p*-value
8A	0.956/–0.015/0.158	0.961/–0.015/0.151	1.116/0.322
8B	0.942/0.010/0.151	0.943/0.010/0.148	0.121/0.737
8C	0.948/–0.030/0.167	0.967/–0.030/0.153	4.543/0.066
8D	0.924/0.087/0.151	0.941/0.087/0.137	2.347/0.641
8E	0.884/0.113/0.143	0.893/0.113/0.134	0.654/0.442
8F	0.918/0.071/0.158	0.951/0.071/0.139	5.480/0.047
8G	0.957/–0.534/0.159	0.960/–0.534/0.153	0.676/0.435
8H	0.942/–0.511/0.151	0.942/–0.511/0.150	0.018/0.898
8I	0.950/–0.547/0.167	0.966/–0.547/0.154	3.615/0.094
8J	0.933/–0.447/0.151	0.943/–0.447/0.141	1.306/0.286
8K	0.888/–0.407/0.143	0.894/–0.407/0.135	0.454/0.520
8L	0.935/–0.476/0.158	0.952/–0.476/0.145	2.810/0.132

**Table 2:** Corresponding modeling results during the late training phase in Fig 8. ** and *** represent significant improvement in modeling predictability of the noise reduction model, with *p* < 0.01 and *p* < 0.001 (false discovery rate [FDR]-corrected).

Figure		Late training phase	
	Gain model	Noise model	Nested model
	R^2^/baseline/noise	R^2^/baseline/noise	F value/*p*-value
8A	0.413/–0.015/0.158	0.952/–0.015/0.096	89.184/<0.001***
8B	0.452/0.010/0.151	0.918/0.010/0.093	45.431/<0.001***
8C	0.326/–0.030/0.167	0.965/–0.030/0.098	147.593/<0.001***
8D	0.248/0.087/0.151	0.882/0.087/0.083	43.087/<0.001***
8E	0.265/0.113/0.143	0.812/0.113/0.080	23.311/0.001**
8F	0.178/0.071/0.158	0.903/0.071/0.085	59.573/<0.001***
8G	0.741/–0.534/0.159	0.970/–0.534/0.118	61.002/<0.001***
8H	0.768/–0.511/0.151	0.946/–0.511/0.115	26.545/<0.001***
8I	0.674/–0.547/0.167	0.978/–0.547/0.119	108.367/<0.001***
8J	0.675/–0.447/0.151	0.943/–0.447/0.107	37.549/<0.001***
8K	0.644/–0.407/0.143	0.880/–0.407/0.102	15.624/0.004**
8L	0.648/–0.476/0.158	0.956/–0.476/0.111	56.283/<0.001**

According to the day-by-day analysis of the behavioral and the P1 data, there was an increase in the maximum P1 response evoked by the focused target during the training interval in which behavioral performance significantly improved (from the first to the second day). Afterward, the maximum P1 response dropped and behavioral performance reached an asymptotic level (after the second day; Figs 2C, 5E and 6D). Using the modeling framework based on SDT described above, we also found that a pure gain model can account for the link between P1 responses and behavior across the first 2 sessions, whereas a noise reduction model was required to better account for the link between the P1 and behavioral data in most of the subsequent training sessions (S2A Fig and S1 Table). Also, note that the modeling data are qualitatively similar for the P1 data with and without baseline subtraction (S2B Fig and S2 Table).

**Fig 9:**
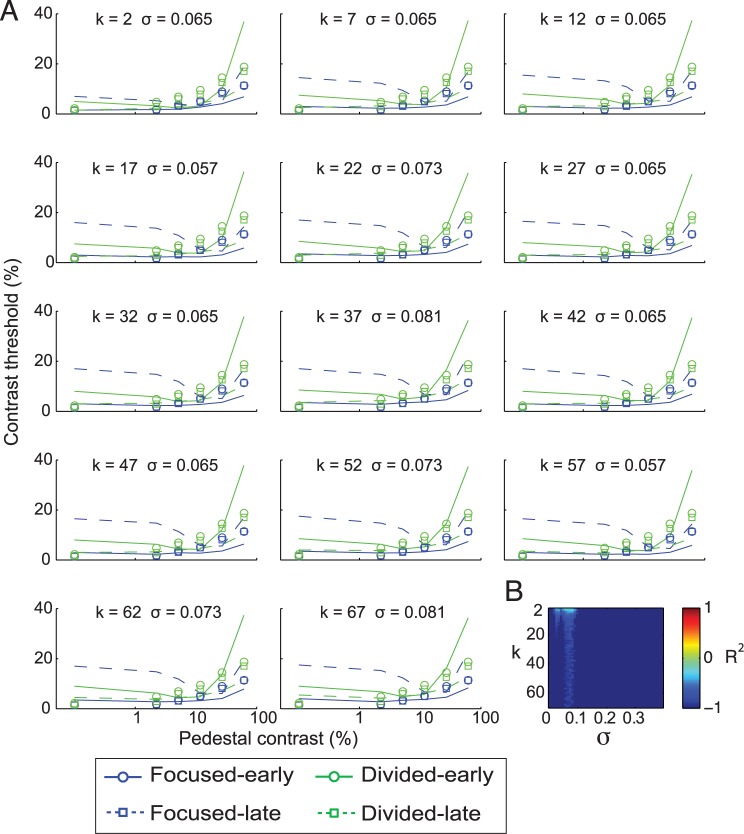
Linking changes in the psychophysical data and the P1 data using the efficient readout model. (A) The psychophysical contrast-discrimination thresholds estimated from the P1 data using the max-pooling rule (Eq 11) with different *k* values and with the noise values that yielded the best fits for those *k* values. (B) Corresponding R^2^ values. Overall, the efficient readout model did not effectively capture the link between behavior and the P1 responses in this data set, as R^2^ values are below 0 for all *k* and noise values. Negative values of the goodness of fit (R^2^) metric indicate that fits are worse than a horizontal fit line. Data are available from the Open Science Framework (https://osf.io/pc7dr/).

In addition to attentional gain and noise reduction, attention is also thought to impact behavior by enhancing the efficiency with which sensory responses are decoded or readout by later sensorimotor and decision-related mechanisms [5,28,61–62]. Therefore, we also considered a variant of an efficient decoding model that is based on a max-pooling rule (Eq 11) to account for behavior during the late training phase [5,28]. However, given that the noise and gain models almost perfectly predicted behavior, using efficient readout actually impaired model predictions in this data set (Fig 9). Note that the negative values of goodness of fit (R^2^) presented in Fig 9 indicate that the efficient decoding model accounted for less variance than a horizontal line. Collectively, these results suggest that training reduced the impact of gain mechanisms and that noise modulations gradually come to play a more dominant role in predicting behavior.

### Training does not attenuate attentional gain of the late positive deflection (LPD) component

**Fig 10:**
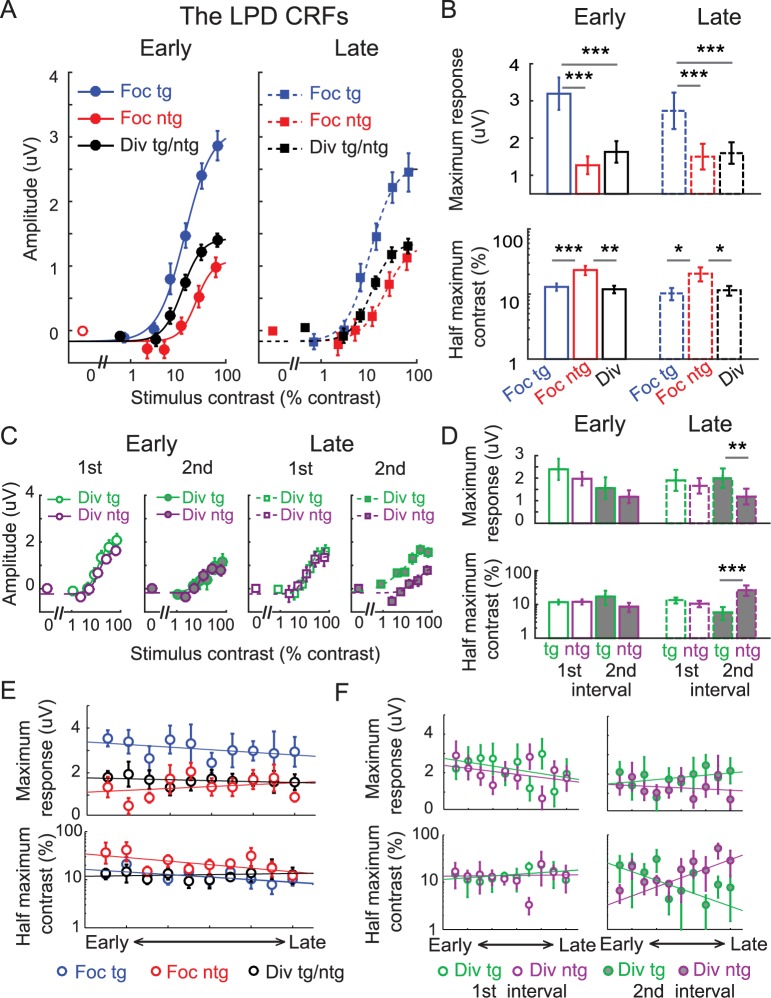
Modulations of the late positive deflection (LPD) component with baseline subtraction. (A) The contrast response function (CRF), based on the amplitude of the LPD component, which was averaged over posterior electrodes from 230–380 ms poststimulus. Focused attention increased the LPD amplitude by a comparable amount across early and late training phases. Error bars represent within-subject SEM. (B) Corresponding maximum response (response at 100% contrast minus baseline) and half-maximum contrast parameters of the CRFs shown in (A). Error bars represent the 68% CIs; *, **, and *** represent significant pairwise comparisons between attention conditions in each training phase, with *p* <0.05, *p* < 0.01, and *p* < 0.001, respectively. (C) The LPD-based CRFs evoked by the divided target nontarget stimuli during the first and second stimulus intervals across the early and late training phases. We observed a separation between the divided target and nontarget responses only during the second stimulus interval of the late training phase, consistent with the idea that the LPD reflects postperceptual decision-making processes that can be enhanced with training [67–68]. Error bars represent within-subject SEM. (D) Corresponding maximum response and half-maximum contrast parameters of the CRFs shown in (C). Error bars represent the 68% CIs; **, and *** represent significant pairwise comparisons between attention conditions in each training phase with *p* < 0.01 and *p* < 0.001, respectively. (E) The day-by-day analysis of the LPD data with baseline subtraction (2 electroencephalography [EEG] sessions or ~1 day per each time bin). Top and bottom panels represent the estimated maximum response and half-maximum contrast parameters. (F) The day-by-day analysis of the LPD data shown in (C) and (D). Error bars in (E) and (F) represent the 68% CIs. Note that the contrast values on the x-axis in (A) and (C) are not exactly the same across target and nontarget conditions because, in the target conditions, we used the averaged contrast values between the pedestal and incremental stimuli. Data are available from the Open Science Framework (https://osf.io/pc7dr/).

In addition to our main analysis of the P1 component, we also examined attentional modulations of another common ERP component referred to as the late positive deflection (LPD or P3), which emerged about 230–380 ms after stimulus onset in central–posterior electrodes (Fig 4). This later ERP component is thought to index postsensory processes such as evidence evaluation during decision making [9,17,63–66]. As shown in Fig 10A, we found robust attentional gain of the LPD in both the early and the late training phases. The maximum response of the LPD-based CRF associated with the focused target condition was significantly higher than the maximum response values in the focused nontarget and divided attention conditions (Fig 10B top, *p*’s < 0.001 for both early and late training phases, resampling tests, 2-tailed). Importantly, we observed no significant changes in the maximum response across early and late training phases in any experimental condition (all *p*’s ≥ 0.208, resampling tests, 2-tailed). In addition, in both early and late training phases, the half-maximum contrast value associated with the focused nontarget condition was significantly higher than those elicited by the focused target (*p* < 0.001 and *p* = 0.027 for early and late training phases, respectively, resampling test, 2-tailed) and the divided attention conditions (*p* = 0.002 and *p* = 0.027 for early and late training phases, respectively, resampling tests, 2-tailed). Moreover, we observed no significant changes in the half-maximum contrast across early and late training phases in any experimental condition (all *p*’s ≥ 0.259, resampling tests, 2-tailed). Consistent with the main results, the day-by-day analysis of the LPD data revealed that attentional modulations of the maximum response of the P1 CRF and the half-maximum contrast were relatively stable throughout training (Fig 10E). Specifically, the slopes associated with the linear fits of the maximum response and the half-maximum contrast were not significantly different than 0 in any condition, indicating no changes in these parameters across training (all *p*’s ≥ 0.090, resampling test, 2-tailed; see Materials and methods).

In the P1 data, there were no changes in response amplitude across divided target and divided nontarget conditions or across the first and second stimulus intervals (Fig 5C and 5D). However, we did observe differences in the LPD responses in the divided target and divided nontarget conditions in the second stimulus interval during the late training phase (*p* = 0.010 and *p* = 0.003 for the maximum response and the half-maximum contrast, respectively, resampling tests, 2-tailed). We did not observe any target or nontarget differences in any other condition (Fig 10C and 10D, all *p*’s ≥ 0.283, resampling tests, FDR-corrected, 2-tailed). In addition, we found a significant 3-way interaction between stimulus type (divided target and nontarget), stimulus interval (first and second), and training (early and late) on the half-maximum contrast of the LPD-based CRFs (*p* = 0.001, resampling test, 2-tailed). Consistent with this finding, the day-by-day EEG analysis showed a significant separation of the half-maximum contrast values between the divided target and divided nontarget conditions across training but only in the second stimulus interval (the slopes of linear fits between the 2 conditions were significantly different from each other, with *p* = 0.016; *p* = 0.722 for the first interval, resampling tests, 2-tailed). We did not observe any significant differences in the maximum response in either of the stimulus intervals (all *p*’s ≥ 0.496). The separation of the responses in the divided target and divided nontarget conditions in the second stimulus interval is consistent with the idea that the LPD indexes postsensory or postperceptual processes [9,17,63–66]. For instance, there should not be a separation of target and nontarget responses during the first stimulus interval because subjects could not yet know whether the left or the right stimulus was the increment target until the second stimulus interval. The separation of the divided target and divided nontarget LPD responses (Fig 10C, 10D and 10F) also suggests that postperceptual processes can improve with training without concomitant changes in early sensory responses (Fig 5C and 5D), consistent with reports from previous studies of perceptual learning [67–68].

**Fig 11:**
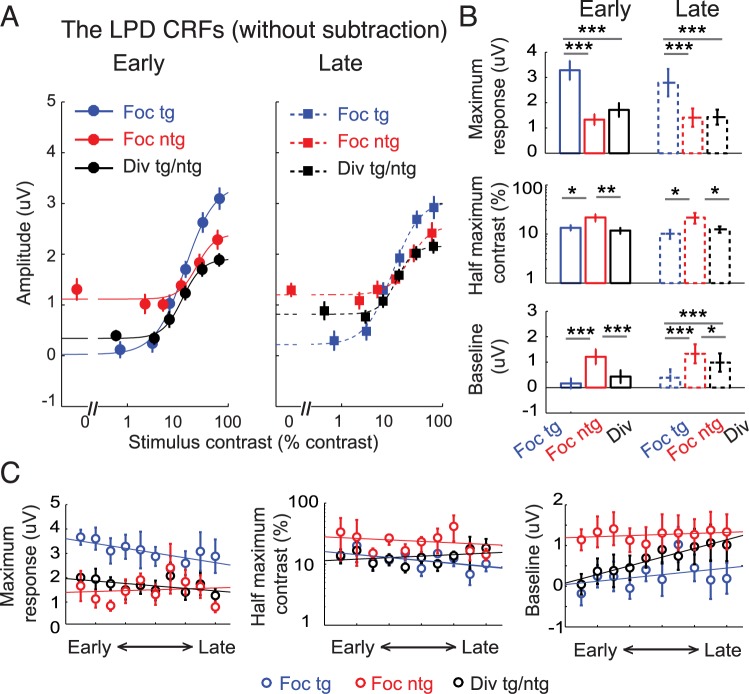
Modulations of the late positive deflection (LPD) component without baseline subtraction. (A) The LPD contrast response functions (CRFs), based on event-related potentials (ERPs) without baseline subtraction. Note that the contrast values on the x-axis are not exactly the same across target and nontarget conditions because in the target conditions, we used the averaged contrast values between the pedestal and incremental stimuli. Error bars represent within-subject SEM. (B) Corresponding maximum response (response at 100% contrast minus baseline), half-maximum contrast, and baseline parameters. Error bars represent the 68% CIs; *, **, and *** represent significant pairwise comparisons between attention conditions in each training phase, with *p* < 0.05 and *p* < 0.001, respectively. (C) The day-by-day analysis of the LPD data without baseline subtraction (2 electroencephalography [EEG] sessions or ~1 day per each time bin). Left, middle, and right panels represent the estimated maximum response, half-maximum contrast, and baseline parameters, respectively. Error bars represent 68% CIs. Data are available from the Open Science Framework (https://osf.io/pc7dr/).

As illustrated in Fig 11A, there were robust modulations of the baseline activity of the LPD component. Thus, for comparison, we also analyzed the LPD data without subtracting the baseline activity levels. A nested model comparison analysis confirmed that allowing the baseline parameter to change freely significantly improved the goodness of fit compared to fixing the baseline parameter across experimental conditions (F(5, 11) = 18.70, *p* <0.001, nested test). In addition, a resampling analysis revealed that the baseline activity of the LPD changed across experimental conditions and across training phases (bottom panel in Fig 11B). In both training phases, baseline activity in the focused nontarget condition was significantly higher than baseline activity in the focused target condition (*p*’s < 0.001 for both training phases) and the divided attention condition (*p* < 0.001 and *p* = 0.014 for early and late training phases, respectively, resampling tests, 2-tailed). We believe that this increase in baseline activity in the focused nontarget (or unattended) condition is due to contamination from responses in the focused target condition, which on average were higher in amplitude than the LPD associated with the focused nontarget condition. This contamination occurred because the LPD is a centrally generated component that is not spatially selective. Thus, unlike the P1, isolating the response uniquely associated with the focused target and with the focused nontarget stimulus was more challenging.

In addition to the attention effect on baseline activity, we also observed a significant increase in the LPD baseline activity across training phases in the divided attention condition (*p* = 0.021) but not in the focused target (*p* = 0.438) or focused nontarget condition (*p* = 0.661, resampling tests, 2-tailed). The day-to-day EEG analysis also revealed similar results, as the slope of the linear fit of the LPD baseline activity in the divided attention condition across time bins was significantly higher than 0, indicating an increase in baseline activity across training days (right panel in Fig 11C; *p* = 0.010, a resampling test, 2-tailed). However, no training-related change in baseline activity was found in the focused target (*p* = 0.450) or focused nontarget condition (*p* = 0.787, resampling tests, 2-tailed). Training effects of the baseline activity in this late ERP component, which is thought to track decision-related processes that are not spatially specific, suggest that improved behavior in the divided attention condition occurred at postperceptual stages of processing rather than in earlier sensory or perceptual stages.

While the LPD results without baseline subtraction revealed some differences in baseline activity, modulations of the maximum response and the half-maximum contrast parameters were consistent with the results reported using the subtraction method (compare Fig 11B and 11C and Fig 10B and 10E). For example, for both early and late training phases, the maximum response associated with the LPD-based CRF elicited by the focused target condition was significantly higher than those associated with the focused nontarget (*p*’s < 0.001 for both early and late training phases) and the divided attention conditions (Fig 11B top, *p*’s < 0.001 for both early and late training phases, resampling tests, 2-tailed). In addition, the half-maximum contrast associated with the LPD-based CRF elicited by the focused nontarget condition was significantly higher than those elicited by the focused target (*p*’s = 0.012 and 0.010 for early and late) and the divided attention conditions (Fig 11B bottom, *p*’s = 0.007 and 0.025 for early and late, respectively, resampling tests, 2-tailed). Importantly, we observed no significant changes in the maximum response and the half-maximum contrast across early and late training phases in any experimental condition (all *p*’s ≥ 0.128, resampling tests, 2-tailed). In addition, the day-by-day analysis of the LPD data showed that attentional modulations of the maximum response and the half-maximum contrast were relatively stable throughout training (left and middle panels on Fig 11C). The slopes associated with the linear fits of the maximum response and the half-maximum contrast in all experimental conditions were not significantly different than 0, indicating no changes in these parameters across days (all *p*’s ≥ 0.086, resampling tests, 2-tailed). Overall, the attentional modulations of the maximum response and the half-maximum contrast persisted across training phases and stood in contrast to the attention effects associated with the P1 results.

## Discussion

Recent studies using quantitative modeling suggest that there are several candidate mechanisms that can link attentional modulations in the visual cortex with attentional modulations of behavior [5,9,28,37,47,51,53]. While some discrepancies between the putative mechanisms are likely due to differences in stimulus display properties, task designs and methods of measuring neural activity [15,45–48,69–71], we show here that another important factor is the duration of training. Similar to previous studies, we found that early in training, attentional gain of the visual P1 component accurately predicted attention-induced behavioral benefits [9,19,31]. However, this attentional gain was abolished later in training, and our SDT-based model suggested that a reduction in noise was required to explain behavior. This later result is consistent with recent reports that highlight the importance of noise reduction by using highly trained nonhuman primates [37,40,43]. Together, these findings suggest that training can change the way that modulations of cortical responses influence behavior, and they underscore the importance of considering training when generalizing empirical observations across methods and species.

In the main analysis that split the data evenly into 2 parts, training had a minimal effect on behavioral performance in the focused attention condition. However, when we examined the behavioral data on a day-by-day basis, we found that there was a change in behavioral performance in the focused attention condition that happened at a much faster rate than in the divided attention condition. As shown in Figs 5E and 6D, we found that the maximum response of the P1-based CRF in the focused-target condition increased from the first to the second day. This increase in the P1 response also tracked a significant improvement in behavioral performance (Fig 2C). However, the maximum P1 response dropped significantly after the second day and behavioral performance started to stabilize. This early modulation followed by a period of relative stability is consistent with reports from a perceptual learning study by Yotsumoto and colleges [60]. In addition, the SDT-based model that we used suggests that attentional gain modulations for visual responses play a substantial role in explaining behavioral performance early on in training but that noise reductions play a dominant role later in training. Recent studies using pharmacological manipulations and electrophysiology in nonhuman primates suggest that attention-related changes in neural gain and neural noise in the visual cortex rely on distinct receptor pathways. Herrero and colleagues found that attentional gain amplification was mediated by muscarinic acetylcholine receptors [72], while attention-related noise reduction was mediated by N-methyl-D-aspartic acid (NMDA) receptor pathways, a critical receptor pathway that is involved in many learning and memory processes, including long-term potentiation [73]. Taken together, these findings suggest that the 2 different temporal dynamics of perceptual learning are facilitated by selective attention and that they may rely on changes in the balance between different receptor pathways that regulate different types of attentional modulations in the visual cortex.

In contrast to the P1 results, consistent gain modulations of the LPD were observed across training. The LPD has been closely linked to postsensory decision-related processing and is associated with factors such as response confidence, task difficulty, and decision times [9,17,63–66,74–75]. Recent EEG studies [63–64] have demonstrated that the LPD also tracks the accumulation of sensory evidence in a manner that is similar to the average response profile of neurons in the lateral intraparietal area and the frontal eye fields in monkeys during decision making [67,76–83]. Collectively, these findings suggest that while attentional modulations of early sensory signals can shift substantially with training, gain modulations of postperceptual processes remain relatively stable over time.

Horizontal eye movements towards an attended target position might play a role in modulating the gain of P1 responses, especially if the eye movements were more frequent after training. However, we view this as unlikely because horizontal eye movements towards the cued location would have attenuated P1 responses by moving the attended stimulus out of the contralateral visual field (see the topographical maps in S1 Fig). In addition, we computed an eye bias score based on the horizontal electrooculography (EOG) signals, and it indicated movement of less than 0.1^o^ visual angle for all individual subjects (see Materials and methods and [54]). We did not observe any differences in the eye bias score across early and late training phases (S3 Fig). Note that while we could control for horizontal eye movements, we could not control for vertical eye movements using the EOG responses. However, the observation of reduced P1 amplitude with training was selective to the focused target condition and not all the other conditions. It is hard to explain this pattern of results based on vertical eye movements because vertical eye movements would have impacted the amplitude of the P1 associated with both the focused target stimuli and the unattended stimuli that were presented on the other side of fixation. In addition, vertical eye movements would likely have shifted the topography of the P1 component to a more posterior site on the scalp, and no such shift was observed (see the topographical maps in S1 Fig).

We implemented a baseline subtraction method to isolate evoked responses associated with a single stimulus from a bilateral stimulus array and to minimize spatially nonspecific anticipatory effects because of the attention cue. This subtraction assumes that EEG responses combine linearly. To address this issue, we controlled stimulus presentation so that every contrast value assigned to a target was counterbalanced with an equal number of nontarget stimuli rendered at all other contrasts. In addition, we observed a qualitatively similar pattern of results with and without the baseline subtraction, particularly with the P1 component. Thus, the subtraction method, and its assumption of linearity, did not spuriously cause the reported changes in P1 amplitude that occurred with training.

Past studies have shown that the contrast of competing stimuli can have a significant impact on the gain of visually evoked responses because of the bottom-up capture of attention and divisive normalization [84–85]. Although evaluating the impact of contrast was not a main goal of the current study, we performed a follow-up analysis to investigate. We found that the attentional gain of the P1 response observed early in training was larger when competing stimuli were rendered at low-to-medium contrast levels (top panels of S4A and S4B Fig). No attentional gain modulation of the P1 was observed when the competing stimuli were rendered at high contrast values. Despite the influence of distractor contrast on P1 attentional gain early in training, the lack of a P1 modulation was consistent across distractor contrast conditions later in training. Future studies could carry out similar experiments with independently measured contrast thresholds for different distractor contrast levels to more formally compare the relative contribution of different neural mechanisms to help better understand interactions between the salience of relevant targets and irrelevant distractors.

Our SDT-based modeling relied on the assumption that attentional modulations of the P1-based CRF primarily reflect neural responses pooled across large populations of neurons in the visual cortex (like LFP). This assumption has been adopted by many previous studies [7,9,12,17–21, 31–32] and has received recent empirical support showing a close connection between the contrast and attentional modulations of spiking activity and those of LFP at the same temporal window as the P1 component (~100 ms poststimulus) [49–50]. However, the relationship between reductions of neuronal noise and the magnitude of the P1 is poorly understood. Therefore, the translation of our modeling results to exact degree of noise reductions in single units must be done with caution. While it seems clear that attentional modulations of the P1 component are decreasing with training, further work using invasive methods will be needed to better describe the changes in single unit attention effects that evolve over training. For example, pharmacological manipulation of different receptor pathways (e.g., muscarinic acetylcholine and NMDA receptors [72–73]) could be carried out to forge a more direct link between attentional modulations in population-based measures and the gain and noise modulations of single units.

In conclusion, our data demonstrate that attentional gain of the visually evoked P1 component plays a prominent role in enhancing perceptual sensitivity early in training, but noise reduction is required after extensive training. In contrast, attentional gain of the LPD component persists throughout training. This pattern is consistent with an attention-related improvement in the efficiency of the transfer of information across the cortical hierarchy, such that earlier stages provide more stable information to downstream areas after training. Most importantly, our data suggest that training does not simply change the magnitude of sensory signals or the magnitude of attentional modulations of sensory signals [86–100]. However, training can qualitatively alter the relationship between attentional modulations of neural responses and behavior, and this observation carries important implications for understanding attention as well as for linking observations collected from different model systems that may employ substantially different amounts of training (c.f. [45]).

## Materials and methods

### Ethics statement

All human participants provided written informed consent as required by the local institutional review board at the University of California, San Diego (UCSD; IRB#110176), and the experiment was conducted under the protocol that followed the Declaration of Helsinki.

### Subjects

Twenty-three neurologically healthy human observers with normal or corrected-to-normal vision were recruited from the UCSD community. Data from 10 subjects were discarded in the main analysis because of failure to complete the experimental protocol (20 EEG sessions). One subject only completed a behavioral training session. Among the other 9 subjects, 1, 1, 1, 3, 1, and 2 subjects voluntarily withdrew after the second, sixth, eighth, 10th, 12th, and 14th EEG sessions, respectively. In addition, 1 of 13 subjects who completed 20 EEG sessions was discarded because of excessive small saccades (>90% of trials). This left 12 subjects in the main analysis (7 female, 20–26 years old, all right-handed). Subjects were compensated at a rate of US$10 and US$15 dollars per hour for behavioral training and EEG sessions, respectively.

### Stimuli and task

Stimuli were presented on a PC running Windows XP using MATLAB (Mathworks Inc., Natick, MA) and the Psychophysics Toolbox (version 3.0.8) [101–102]. Participants were seated 60 cm from the CRT monitor (which had a grey background of 34.51 cd/m^2^, 60Hz refresh rate) in a sound-attenuated and electromagnetically shielded room (ETS Lindgren).

Subjects performed a 2IFC contrast discrimination task (Fig 1A). Each trial started with a colored precue instructing subjects where to attend on each trial. A red cue corresponded to the lower left quadrant, a blue cue corresponded to the lower right quadrant (focused attention), and a green cue indicated that subjects should attend to both lower quadrants (divided attention). The focused attention cue was 100% valid and always indicated the location of the target, whereas the divided attention cue indicated that the target was equally likely to be presented in the left or the right quadrant. The precue appeared for 500 ms, followed by a 400–600ms blank interstimulus interval (ISI). This ISI was followed by 2 successive stimulus presentations (the first and second stimulus intervals), with each interval containing a pair of sinusoidal Gabor stimuli (spatial frequency, 1.04 cycles per degree; SD of Gaussian window, 1.90°) located in the lower left and right quadrants (±8.58° and −7.63° from the horizontal and vertical meridians, respectively). Each pair of stimuli appeared for 300 ms, followed by a 600–800 ms ISI (pseudorandomly jittered from a uniform distribution). The pedestal contrasts of the Gabor stimuli were randomly selected from 6 values: 0%, 2.24%, 5.13%, 11.75%, 26.92%, and 61.66% Michelson contrasts. Contrast values, except for 0%, were jittered ±0.01 log contrast from the mean contrast values. The orientations of the left and right Gabors were identical within each trial, and the orientation value on a given trial was randomly drawn from a uniform distribution. During 1 of the 2 stimulus intervals, a contrast increment (Δc) was added to either the left or the right Gabor stimulus for the entire duration of that interval. After the second stimulus interval, the postcue appeared to inform subjects whether the left (a red cue) or the right stimulus (a blue cue) contained this contrast increment target. Subjects reported whether the increment occurred during the first or second stimulus interval. They were told to prioritize accuracy and there was no response deadline.

On the first day, subjects participated in a ~2.5-hour behavioral training session in which a staircase procedure (3 down, 1 up) was applied to estimate the contrast discrimination thresholds for each attention condition and each pedestal contrast level (see a similar method in [9, 56]). These thresholds were then used in the first EEG session. Subjects completed 20 EEG sessions (2–3 days a week, 1–2 EEG sessions per day, with 2 sessions completed on an average of 12.08 days across subjects). Each EEG session contained a total of 8 experimental blocks and contained 288 trials, in which all experimental conditions were counterbalanced: 2 (attention conditions: focused, divided) × 2 (target locations: left, right) × 2 (target intervals: first, second) × 6 (pedestal contrast levels of target) × 6 (pedestal contrast levels of nontarget). The contrast threshold (Δc) for each attention condition and each target pedestal contrast was adjusted after each EEG session so that accuracy was maintained at ~76% (d’ = ~1) across all experimental conditions. Across the 12 subjects, the average time elapsed between the initial behavioral training session and the first EEG session, between the first and the 11^th^ EEG sessions (early training phase), and between the 11^th^ and the last EEG sessions was 3.33 ± 0.66, 17.41 ± 2.31, and 11.50 ± 1.12 days (mean ± SEM), respectively (Fig 1B).

### Behavioral analysis

Contrast discrimination thresholds were measured at ~76% hit rate (d’ = ~1) for all attention conditions (focused and divided attention), training phases (early and late), and stimulus pedestal contrasts (0% to 61.66%). In the main behavioral analysis, we divided the data into 2 learning phases (the first 10 EEG sessions for the early phase and the last 10 EEG sessions for the late phase) to match the main EEG analysis, in which we sought to obtain ~400 trials for each data bin as suggested by Luck [54]. The within-subject SEM of the data for each contrast level was calculated by removing the mean value of each attention and training condition from the individual subject data before computing the SEM [103]. Three-way repeated measures ANOVAs with within-subject factors of attention condition, training phase, and pedestal contrast were performed to test the main effect of each of these factors and their interactions on contrast discrimination thresholds. Post hoc paired *t* tests were then used to examine attention effects and learning effects on the contrast discrimination threshold data for each pedestal contrast level, and multiple comparisons were corrected by the FDR method with the α value of 0.05 [104]. We used 1-tailed statistics here under the assumption that the behavioral performance should improve with attention and training.

To examine training effects on a finer temporal scale, we also performed a day-by-day behavioral analysis in which the contrast discrimination thresholds were divided into 10 time bins (2 EEG sessions per bin or ~1 day per bin since subjects completed 2 EEG sessions on most days). We could only subdivide the data into 10 bins because after artifact rejection, there were often missing values in the ERP data from a single EEG session for some of the 24 experimental conditions (2 attention conditions x 2 stimulus types x 6 contrast levels, with 11 out of 20 EEG sessions having at least 1 missing value). Repeated-measures 1-way ANOVAs with time as a within-subject factor were performed to test the effect of training on the contrast discrimination thresholds separately for stimuli of different pedestal contrast levels in both focused attention and divided attention conditions. Multiple comparisons were corrected by the FDR method with the α value of 0.05 [104]. To statically evaluate day-by-day changes in discrimination thresholds, post hoc paired *t* tests were performed to examine the difference in the threshold on data between the first and the second days and between the second day and the remaining days. We used 1-tailed statistics here under the assumption that behavioral performance should improve with training. Multiple comparisons were corrected by the FDR method with the α value of 0.05 [104].

### EEG preprocessing and analysis

We recorded EEG data with a 64 + 8 channel Biosemi ActiveTwo system at a 512-Hz sampling rate. All signal offsets from the CMS-DRL reference were maintained below 20 uV. We employed EEGlab11.0.3.1b [105] and custom MATLAB scripts to preprocess the EEG data offline. First, we rereferenced the continuous EEG data to the mean of the 2 mastoid electrodes and applied 0.25-Hz high-pass and 55-Hz low-pass Butterworth filters (third order). Second, the data were segmented into epochs extending from 500 ms before to 3,500 ms after the trial onset. Third, prominent eye-blink artifacts were first rejected by independent component analysis [106]. We then discarded epochs contaminated by residual eye blinks and vertical eye movements (more than ±80–120 μV deviation from 0, with thresholds chosen for each individual subject), horizontal eye movements (more than ±75–90 μV deviation from 0), excessive muscle activity, or drifts using threshold rejection and visual inspection (11.23% of trials ± 1.74% SEM).

Lastly, the data were aligned to the stimulus onset and baseline corrected based on the mean response from 0–200ms before stimulus onset. For all individual subjects, eye bias scores computed by the difference between averaged horizontal EOG contralateral and ipsilateral to the stimulus of interest divided by 2 were less than 1.6 μV, corresponding to less than 0.1^o^ visual angle, which is a standard criterion used in ERP studies [54]. Moreover, no difference in eye bias scores were observed across early and late training phases (S3 Fig). These results support the notion that any residual horizontal eye movements did not contaminate attention-related and training-related changes in ERPs.

The artifact-free EEG data were then sorted into the following bins: 2 attention conditions (focused and divided attention) x 2 stimulus types (target and nontarget) x 2 training phases (early and late) x 6 stimulus contrast levels x 2 stimulus intervals (first and second) x 2 stimulus locations (left and right). The stimulus-locked ERPs were then computed by averaging the EEG data in each bin. To extract ERPs evoked by the stimulus of interest (i.e., subtract out responses evoked by stimuli paired with the stimulus of interest) and minimize confounds from any anticipatory effect from the cue, we subtracted the ERPs evoked by the pedestal 0% contrast stimulus (i.e., when no stimulus was present in the contralateral visual field with respect to a given EEG electrode) from the ERPs in all other conditions [9,31,59] (Fig 3). Thus, the response that was subtracted should be interpreted as “the mean response evoked by an ipsilateral stimulus when no stimulus was presented in the contralateral visual field”, and this served to help isolate the ERP specifically associated with the presentation of a contralateral stimulus. It is critical to isolate the CRFs evoked by the stimuli of interest (the focused target, focused nontarget, divided target, and divided nontarget conditions) from the stimuli that were simultaneously presented on the other side of the display (e.g., if the stimulus of interest was a focused target, then the stimulus that was paired with it would be a focused nontarget). Thus, subtracting out the small response evoked by the ipsilateral stimulus helps to improve the spatial selectivity of the ERP responses. Moreover, without subtracting this 0% contrast ERP out, it is possible that any attentional modulations would be confounded by cue-related and nonspatially selective anticipatory responses rather than attentional modulations of stimulus-evoked responses (i.e., changes in arousal but not changes in selective spatial attention). This issue has been addressed in a similar manner in many previous studies that use EEG and fMRI [9,28,31,59]. However, we also include the results without this baseline subtraction to validate the linearity assumption of this method (Figs 6, 8G–8L and 11; see detailed methods in later paragraphs).

The mean amplitude of the visual P1 component from 80–130 ms post-stimulus was computed across the contralateral-posterior electrodes, where the P1 mean amplitude averaged across all experimental conditions was the highest (PO7, P5, and P7 for the left hemisphere and PO8, P6, and P8 for the right hemisphere). The selected temporal window was based on previous ERP studies of visual attention [7,9,12,17–21,31–32], and the 50-ms window size is suggested as the standard by Luck [54]. The mean P1 amplitude was then plotted as a function of stimulus contrast to yield the P1-based CRF separately for each attention condition, each stimulus type, and each training phase. In the main EEG analysis, we divided the data into 2 training phases (10 EEG sessions for each phase). This division of the data into 2 phases was motivated by the desire to have ~400 trials in each experimental condition as suggested by Luck [54]. However, as a supplement to this planned analysis, we also conducted an additional analysis of the EEG data collected on each day and thus divided into 10 bins (with ~2 EEG sessions for each time bin or ~1 day per bin given that subjects completed 2 EEG sessions on most days). On the x-axis of the CRF, the stimulus contrast values for the focused and divided nontargets were fixed at 0%, 2.24%, 5.13%, 11.75%, 26.92%, and 61.66% Michelson contrasts. However, since the target sequence contained both pedestal and increment stimuli, we used the averaged contrast values between the 2 stimuli for plotting the CRFs in the focused-target and divided-target conditions. The within-subject SEM of the data for each contrast level was calculated using the Loftus and Masson method [103] in which the mean value between attention, stimulus type, and training conditions was removed from individual data before computing the SEM for each contrast value. Next, the P1 data were bootstrapped by resampling subjects, with replacement, 10,000 times. In each bootstrap iteration, the CRF data for each attention condition, stimulus type, and training phase were fit with a Naka–Rushton equation:

$$R(c)=G_{r}\frac{c^{q}}{c^{q}+{G_{c}}^{q}}+b,$$ (1)

where *R(c)* is the P1 amplitude as a function of stimulus contrast, *G*_*r*_ is a multiplicative response gain factor that controls the vertical shift of the CRF, *G*_*c*_ is a contrast gain factor that controls the horizontal shift of the CRF, *b* is the response baseline offset, and *q* is the exponent that controls the speed at which the CRF rises and reaches asymptote. Given that past EEG studies of spatial attention have consistently reported no changes in response baseline of EEG-based CRFs [3,9,10,13–14,33], and in the present study the evoked response to 0% contrast stimuli was subtracted from all trials (such that the ERP was flat on 0% contrast trials) *b* was fixed as the average of the minimum amplitude across all experimental conditions. We then used a least square error estimation method (fminsearch function in MATLAB) to estimate the maximum response (the response at 100% contrast minus baseline), the half-maximum contrast (contrast at which the response reached half-maximum), and the exponent (*q*) parameters. Since in many experimental conditions the CRFs did not fully saturate at the maximum contrast level (100%), we constrained the fitting procedure so that the maximum response value could not exceed 1.5 times as large as responses at the 61.66% contrast value (the highest contrast in the stimulus set). *G*_*r*_ *and G*_*c*_ were constrained so that they could not be less than 0 and 1, respectively. The exponent *q* was also constrained within a range of –10 to 10. We used the 30% contrast value (about half of 61.66% contrast) as the initial seed value for *G*_*c*_, the difference between maximum and minimum responses as the seed value for *G*_*r*_, and 1 and 5 for the seed values of the exponent *q* when fitting the CRFs based on the P1 and the LPD (see below for LPD), respectively. The initial seed values for the exponent *q* were adopted from the estimated values based on a previous study [9].

For each training phase, we first tested the effect of attention cueing (cued versus uncued) on the maximum response of the P1-based CRF in the focused attention condition. To do so, we computed the bootstrap distribution of the difference between the estimated fit parameters in the focused target (i.e., attended) and the focused nontarget (i.e., unattended) conditions and computed the percentage of values in the tail of this compiled distribution that were larger or smaller than 0 (2-tailed to be conservative). Next, we tested the effect of attention cue type (focused versus divided attention) on the maximum response of the P1-based CRF by computing the bootstrap distribution of the difference between the estimated fit parameters in the focused target and the divided attention conditions. We then computed the percentage of values in the tail of this compiled distribution that were larger or smaller than 0 (2-tailed). Note that in the divided attention condition, we collapsed the data between the divided target and divided nontarget conditions since there was no difference in the P1 amplitude associated with these 2 conditions (see Fig 5C). For the P1 maximum response, we observed a robust attentional gain modulation early in training, but this gain modulation disappeared after training (Figs 5 and 6). We statistically evaluated this observation by examining the interaction between training (early versus late) and attention cue type (focused versus divided attention), and we compared the distribution of the difference between the focused target and the divided attention conditions during the early training phase and the distribution of the difference during the late training phase. We then computed the percentage of values for which the early-training-phase distribution was larger than the late-training-phase distribution (1-tailed because of the assumed direction of the interaction). Lastly, we evaluated whether the maximum response values in the focused target, focused nontarget, and divided attention conditions changed across training phases. To do so, for each experimental condition, we computed the bootstrap distribution of the difference of the estimated fit parameter in each condition across early and late training phases and calculated the percentage of values in the tail of this compiled distribution that were larger or smaller than 0 (2-tailed). The same analysis was then performed on the half-maximum contrast values associated with the P1-based CRFs.

For the LPD component, the mean amplitude from 230–380 ms poststimulus was computed across the posterior and posterior–occipital electrodes (P5, P7, PO7, P1, Pz, P2, P6, P8, and PO8). This analysis window and electrodes were selected based on the broad activation of the LPD amplitude and averaged across all experimental conditions and stimulus contrast levels. Also note that the analysis windows of both P1 and LPD components were chosen to minimize contamination from the negative-going N1 component that emerged ~150–200 ms poststimulus (see the zoom-in ERP traces for the P1 and LPD components in Fig 4B and 4C, in which minimal negative potentials were observed across these windows). The same bootstrapping, fitting, and statistical analyses described above were also performed on the LPD data.

For comparison, we also analyzed the P1 and the LPD data without baseline subtraction (see results in Figs 6 and 11). First, we obtained the P1 and LPD components in the same electrodes and the same temporal windows as described above and plotted the CRFs based on the P1 and LPD mean amplitudes. However, the baseline subtraction described above was not implemented. Next, we fit the Naka–Rushton equation (Eq 1) to characterize the CRFs, but this time we included an additional free parameter to account for baseline differences between conditions. We then performed a nested model comparison to assess the goodness of fit between the model that allowed baseline parameters to change freely (baseline-free model) and the model that fixed the baseline parameter across all experimental conditions (baseline-fixed model) using the following equation:

$$\frac{R_{baseline-free}^{2}-R_{baseline-fixed}^{2}}{{Df}_{1}}/\frac{1-R_{baseline-free}^{2}}{{Df}_{2}}$$ (2)

where $R_{baseline-free}^{2}$ and $R_{baseline-fixed}^{2}$ were obtained from the fits of the baseline-free and baseline-fixed models (full and reduced models), respectively. *Df*_1_ is the number of free parameters in the full model (24: 6 *b’s*, 6 *G*_*r*_*’s*, 6 *G*_*c*_*’s*, and 6 *q’s* for the focused target, focused nontarget, and divided attention conditions in the early and late training phases) minus the number of free parameters in the reduced model (19: 6 *G*_*r*_*’s*, 6 *G*_*c*_*’s*, and 6 *q’s* for the focused and divided and target and nontarget conditions in the early and late training phases and 1 *b* shared across all 6 experimental conditions). *Df*_2_ is the number of observations (36: 6 contrast levels times 6 experimental conditions) minus the number of free parameters in the full model (24) minus 1. The *F* distribution was used to estimate the probability that the full model differed significantly from the reduced model. For the P1 data, the baseline-free model was not significantly better than the baseline-fixed model (see Results), so we only evaluated the significance of the best fit parameters estimated using Eq 1 with a fixed baseline parameter. On the other hand, the baseline-free model was significantly better for the LPD data, so we reported statistical results using a version of Eq 1 with a freely optimized baseline parameter.

For the daily analysis of the P1 and LPD data (with and without baseline subtraction), the resampling and refitting routines followed the same approach as described above, except that they were performed separately across 10 instead of 2 time bins (~1 day per bin or 2 EEG sessions). To examine whether the fit parameters associated with the P1-based and LPD-based CRFs changed with training, in each resampling and refitting iteration, we fit the estimated CRF parameters across 10 time bins with linear functions to obtain the slopes of those linear fits. Then, we computed the percentage of values in the tails of these compiled distributions that were larger or smaller than 0 (2-tailed).

### Modeling methods

We adopted a previously established model based on SDT [5,9,28,47,52–53] to determine the degree to which attentional gain and noise reduction were needed to explain the relationship between attentional modulations in the psychophysical and ERP data during the early and late training phases. This modeling framework is based on the assumption that perceptual sensitivity (d’) is limited by the differential mean response: *R(c+Δc(c))-R(c)* or ΔR, evoked by 2 different stimuli (i.e., standard and test stimuli) divided by the trial-by-trial variability of those responses (*σ*):

d’=ΔR/σ (3)

where *R* is the hypothetical CRF estimated using the Naka–Rushton equation (Eq 1). With the combination of the d’ and Naka–Rushton equations, the contrast discrimination thresholds could be estimated based on the derivative (or slope) of the CRF as expressed in the following equation:

Δc=ΔR/(dR/dc), (4)

where *dR/dc* is the derivative of the underlying CRF [52].

According to the attentional gain model, attention-induced reductions in contrast discrimination thresholds can be fully explained by an increase in the slope of the ERP-based CRF (*dR/dc*), under the assumption that the neuronal noise (*σ*) is constant (gain model). In the case where the amount of increase in the CRF slope is insufficient to explain shifts in the psychophysical TvC functions, the σ parameter must be reduced to explain changes in psychophysical contrast thresholds (noise model).

We applied this model using the following procedure: we first estimated the psychophysical TvC functions for the divided attention and focused attention conditions for both the early and late training phases using a polynomial function (power = 3) with least square error estimation methods (fminsearch function in MATLAB). Next, we used the combination of the Naka–Rushton and d’ equations to simulate the CRFs based on the P1 amplitude in the divided attention condition during the early training phase in which the behavioral performance was the poorest. Note that in the main analysis, the divided attention data were based on the average between the divided target and divided nontarget conditions across 2 stimulus intervals. However, we also performed modeling in which the divided attention data were based on only the divided target data or on only the divided nontarget data, during the first stimulus interval only, and with and without baseline subtractions. Importantly, all these different analyses yielded consistent results.

The fitting routine started by setting the first point of the estimated CRF (*c*_*0*_ = 0%) to be a baseline parameter (*b*) as the following:

$$R\left(c_{0}\right)=b$$ (5)

The next contrast (c_1_) was then defined as the following:

$$c_{1}=c_{0}+\Delta c_{0}$$ (6)

where *Δc*_*0*_ is the contrast threshold at 0% contrast. Accordingly, the response at *c*_*1*_ was estimated using the d’ equation (Eq 2) as follows:

$$R\left(c_{1}\right)=b+\sigma ,$$ (7)

given that *d’* = 1. The next contrast was defined the same way as the following:

$$c_{i}=c_{i-1}+\Delta c_{i-1}$$ (8)

where *i* is the current iteration that is > 1. The response at *c*_*i*_ was then estimated as the following:

$$R\left(c_{i}\right)=R\left(c_{i-1}\right)+\sigma$$ (9)

These last 2 steps (Eqs 8 and 9) were continued until the full CRF was estimated. The baseline and noise parameters (*b* and *σ*) were optimized by minimizing the least-squares errors between the observed and the predicted CRFs based on the P1 amplitude in the divided target condition. To test if attentional gain changes in the P1 CRFs in each training phase could account for changes in the TvC functions, we estimated the P1 CRFs in the focused target condition in the early and late training phases using the modeling routine described above but with the *b* and σ parameters fixed based on the values obtained from the divided attention condition during the early training phase. Next, we tested if allowing changes in the noise parameter in the focused target condition in the early and late training phases could significantly improve the prediction of the model based on SDT. To achieve this, we estimated the P1 CRFs in the focused-target condition as described above, except that we allowed the σ parameter to vary freely to find the best fit. The R^2^ value obtained from the gain model with the σ parameter fixed across the divided target and focused target conditions (reduced model) was then compared with the R^2^ value obtained using the noise reduction model in which the σ parameter was also allowed to vary freely across attention conditions (full model). This comparison was done using a nested F test:

$$F({Df}_{1},{Df}_{2})=\frac{R_{noise}^{2}-R_{gain}^{2}}{{Df}_{1}}/\frac{1-R_{noise}^{2}}{{Df}_{2}}$$ (10)

where $R_{gain}^{2}$ and $R_{noise}^{2}$ were obtained from the fits of the attentional gain and noise modulation models (reduced and full models), respectively. *Df*_1_ is the number of free parameters in the full model (3: *σ* for focused attention, *σ* for divided attention, and *b* shared across attention conditions) minus the number of free parameters in the reduced model (2: *σ* and *b* shared across attention conditions). *Df*_2_ is the number of observations (12: 6 contrast levels times 2 attention conditions) minus the number of free parameters in the full model (3) minus 1. The *F* distribution was used to estimate the probability that the full model differed significantly from the reduced model.

To test if the gain model or the noise model could better account for the behavioral and the P1 data on a day-by-day basis, we employed a similar modeling framework based on SDT as described above. The modeling routine started by optimizing the noise and baseline parameters to yield the best fit for the P1-based CRF evoked by the focused target condition on the first day using Eqs 5–9 with least square error estimation methods, given the level of behavioral contrast thresholds in the focused attention condition in the same day. Next, given the noise and baseline parameters from that day, we estimated how much gain accounted for the relationship between the P1 data and behavioral data in the following time bins (the second to 10^th^ days) and whether optimizing noise parameters could better account for this relationship using the nested test F-test (Eq 10). Multiple comparisons were corrected using the FDR method with the α value of 0.05 [104].

In addition to the gain and noise models based on SDT, we also adopted a variant of an efficient decoding model to see how well it could explain the link between attentional modulations in the P1 component and behavioral data across training stages. To start the procedure, we first fit the neural CRFs based on the P1 amplitudes with the Naka–Rushton equation (Eq 1 and see the fitting procedure below the equation). Since the model requires all responses to be positive values (because of the *k* exponent in the max-pooling rule; see Eq 11 below), we subtracted the baseline values from the interpolated CRFs of all attention conditions and training stages. Next, for each attention condition of each training phase, we simulated the performance of an ideal observer in 72,000 randomly generated trials, which consisted of 12,000 trials of each of the 6 levels of target pedestal contrasts. These 12,000 trials included 2,000 trials of each of the 6 levels of nontarget contrasts. For each simulated trial, we determined the response of each stimulus type (target or nontarget) and stimulus interval (the interval that contains the test contrast or pedestal contrast) as a random draw from a Gaussian distribution with mean values equal to the mean amplitude of the interpolated P1 CRFs at the corresponding contrast value. The SD of the Gaussian distribution is the noise parameter in the d’ equation (Eq 3) and it was varied from 0.001 to 0.393 in fifty 0.008-unit incremental steps. Next, the target and nontarget related responses (*R*_*tg*_ and *R*_*ntg*_) were pooled into a single response (*R*_*p*_) using the max-pooling equation [5,28]:

$$R_{p}=\frac{\sqrt[{k}]{R_{tg}^{k}+R_{ntg}^{k}}}{2}$$ (11)

where *k* is an exponent that weights responses to each stimulus in a given interval. Under the assumption that an ideal observer would select the interval that contained a larger pooled response as the interval that contained the incremental target stimulus, we searched for the contrast increment value that yielded 76% accuracy rate across the 12,000 simulated trials at each pedestal contrast level. Here, *k* was varied from 2 to 70 in sixty-nine 1-unit incremental steps.

## Supporting information

S1 Table Corresponding modeling results in S2A Fig. * and *** represent significant improvement in modeling predictability of the noise reduction model with p <0.05 and p <0.001 (FDR-corrected). ^V^ indicates that the noise model predicts a reduction in the noise parameter.
(PDF)

S2 Table Corresponding modeling results in S2B Fig. ** and *** represent significant improvement in modeling predictability of the noise reduction model with p <0.01 and p <0.001 (FDR-corrected). ^V^ indicates that the noise model predicts a reduction in the noise parameter.
(PDF)

S1 Fig Topographical maps depicting the visual P1 component and the LPD component during early and late training phases. The left and right sides of the head model represent electrodes ipsilateral and contralateral to the stimulus of interest, respectively.
(TIF)

S2 Fig The day-by-day SDT modeling results. The day-by-day SDT modeling results for the P1 data in the focused target condition with (A) and without baseline subtraction (B), where we found the rising and falling of the maximal response amplitude before and after the behavioral performance reached the saturation point (after the 2^nd^ time bin). The increase in the P1 maximal response (Figs 5E & 6D) that occurred in parallel with the fast improvement in behavioral performance from the 1^st^ and 2^nd^ time bins (Fig 2C) could be sufficiently explained by the gain model without the need to optimize the noise parameter. However, in later time bins when the P1 maximal response reduced back to the same level as the 1st time bin and when the behavioral performance reached the saturation point, the noise model yielded better predictions than that the gain model and it suggests that noise parameters after the 2nd time bin need to be reduced to account for the relationship between behavioral performance and the P1 data. See corresponding model parameters in S1 Table and S2 Table. Also note that any negative value of the goodness of fit (R^2^) show that the model is worse than a horizontal fit line.
(EPS)

S3 Fig Horizontal EOG data. (A) Grand-averaged eye bias scores for the focused/divided target/nontarget conditions. (B-C) Corresponding t and p values describing comparisons of eye bias scores across early and late training phases. We found no difference in eye bias scores across training phases at any time point from -100 to 600ms relative to the stimulus onset. Note that we applied an additional 22-Hz low-pass filter to plot the EOG data in (A) since the EOG data are relatively noisier than the ERP data due to the higher impedance of the EOG electrode but the statistic was performed on the data with the 55-Hz low-pass filter to be conservative.
(EPS)

S4 Fig The P1-based CRFs in the contralateral posterior-occipital electrodes sorted by the contrast of the ipsilateral stimuli. (A) The baseline-subtracted P1-based CRFs in the contralateral posterior-occipital electrodes sorted by the contrast of the ipsilateral stimuli. Note that the contrast values on the x-axis are not exactly the same across target and nontarget conditions because, in the target conditions, we used the averaged contrast values between the pedestal and incremental stimuli. (B) Corresponding maximal response parameters shown in (A). Overall, we found that the attentional modulations in the P1 maximal response during early training phases were primarily driven by trails where the ipsilateral stimuli had low-to-medium contrast levels, but not trials where the ipsilateral stimuli had high contrast levels. In the later training phases, we observed no attentional modulations in any of ipsilateral contrast conditions. (C) Note that we did not measure the psychophysical contrast threshold separately for trials of different nontarget contrasts; therefore, the accuracy varied across nontarget contrast conditions. Error bars in (A) and (C) represent within-subject SEM. Error bars in (B) represent the 68% CIs. ^#^, **, and *** in (B) represent marginal and significant increases in the P1 maximal responses between focused target and the average between in the focused nontarget and divided attention conditions with p< 0.10, p <0.05 and p<0.001, respectively.
(EPS)

## Abbreviations

2IFC: 2-interval forced choice
CRF: contrast response function
EEG: electroencephalography
EOG: electrooculography
ERP: event-related potential
FDR: false discovery rate
LFP: local field potential
LPD: late positive deflection
NMDA: N-methyl-D-aspartic acid; n.s., not significant
SDT: signal detection theory
TvC: threshold versus contrast
