# Supplementary material for: Two different mechanisms support selective attention at different phases of training
Source: PLoS Biol. 2017 Jun 27;15(6):e2001724. doi: 10.1371/journal.pbio.2001724 (PMC5486967; doi:10.1371/journal.pbio.2001724)
Supplement: S1 Table — * and *** represent significant improvement in modeling predictability of the noise reduction model with p <0.05 and p <0.001 (FDR-corrected). V indicates that the noise model predicts a reduction in the noise parameter. (PDF) [file pbio.2001724.s001.pdf]

Data conditions (day to day)	P1 with baseline subtraction		
	Gain model	Noise model	Nested model
	R^2/baseline/noise	R^2/baseline/noise	F value/ p value
1st to 2nd	0.885/0.363/0.169	0.897/0.363/0.185	0.936/0.362
1st to 3rd	0.224/0.363/0.169	0.763/0.363/0.067[v]	33.347/<0.001***
1st to 4th	0.559/0.363/0.169	0.749/0.363/0.123[v]	6.060/0.039*
1st to 5th	-2.003/0.363/0.169	0.261/0.363/0.005 [v]	24.507/0.001***
1st to 6th	-0.135/0.363/0.169	0.772/0.363/0.067 [v]	31.781/<0.001***
1st to 7th	0.095/0.363/0.169	0.154/0.363/0.116[v]	0.556/0.477
1st to 8th	-0.282/0.363/0.169	0.750/0.363/0.065[v]	32.993/<0.001***
1st to 9th	-1.255/0.363/0.169	0.460/0.363/0.023[v]	25.394/0.001***
1st to 10th	-0.431/0.363/0.169	0.720/0.363/0.053[v]	32.893/<0.001***
