# Supplementary material for: Two different mechanisms support selective attention at different phases of training
Source: PLoS Biol. 2017 Jun 27;15(6):e2001724. doi: 10.1371/journal.pbio.2001724 (PMC5486967; doi:10.1371/journal.pbio.2001724)
Supplement: S2 Table — ** and *** represent significant improvement in modeling predictability of the noise reduction model with p <0.01 and p <0.001 (FDR-corrected). V indicates that the noise model predicts a reduction in the noise parameter. (PDF) [file pbio.2001724.s002.pdf]

Data conditions (day to day)	P1 without baseline subtraction		
	Gain model	Noise model	Nested model
	R^2/baseline/noise	R^2/baseline/noise	F value/ p value
1st to 2nd	0.864/-0.510/0.169	0.898/-0.510/0.195	2.678/0.140
1st to 3rd	0.508/-0.510/0.169	0.899/-0.510/0.105^{v}	31.119/<0.001***
1st to 4th	0.604/-0.510/0.169	0.742/-0.510/0.130^{v}	4.236/0.074
1st to 5th	0.872/-0.510/0.169	0.893/-0.510/0.153^{v}	1.564/0.246
1st to 6th	0.752/-0.510/0.169	0.936/-0.510/0.123^{v}	21.781/0.002**
1st to 7th	0.080/-0.510/0.169	0.280/-0.510/0.123^{v}	2.220/0.175
1st to 8th	0.375/-0.510/0.169	0.789/-0.510/0.103^{v}	15.676/0.004**
1st to 9th	0.700/-0.510/0.169	0.874/-0.510/0.123^{v}	11.115/0.010**
1st to 10th	0.467/-0.510/0.169	0.886/-0.510/0.099^{v}	29.444/<0.001***
