# Supplementary figures and images for: Two different mechanisms support selective attention at different phases of training
Source: PLoS Biol. 2017 Jun 27;15(6):e2001724. doi: 10.1371/journal.pbio.2001724 (PMC5486967; doi:10.1371/journal.pbio.2001724)

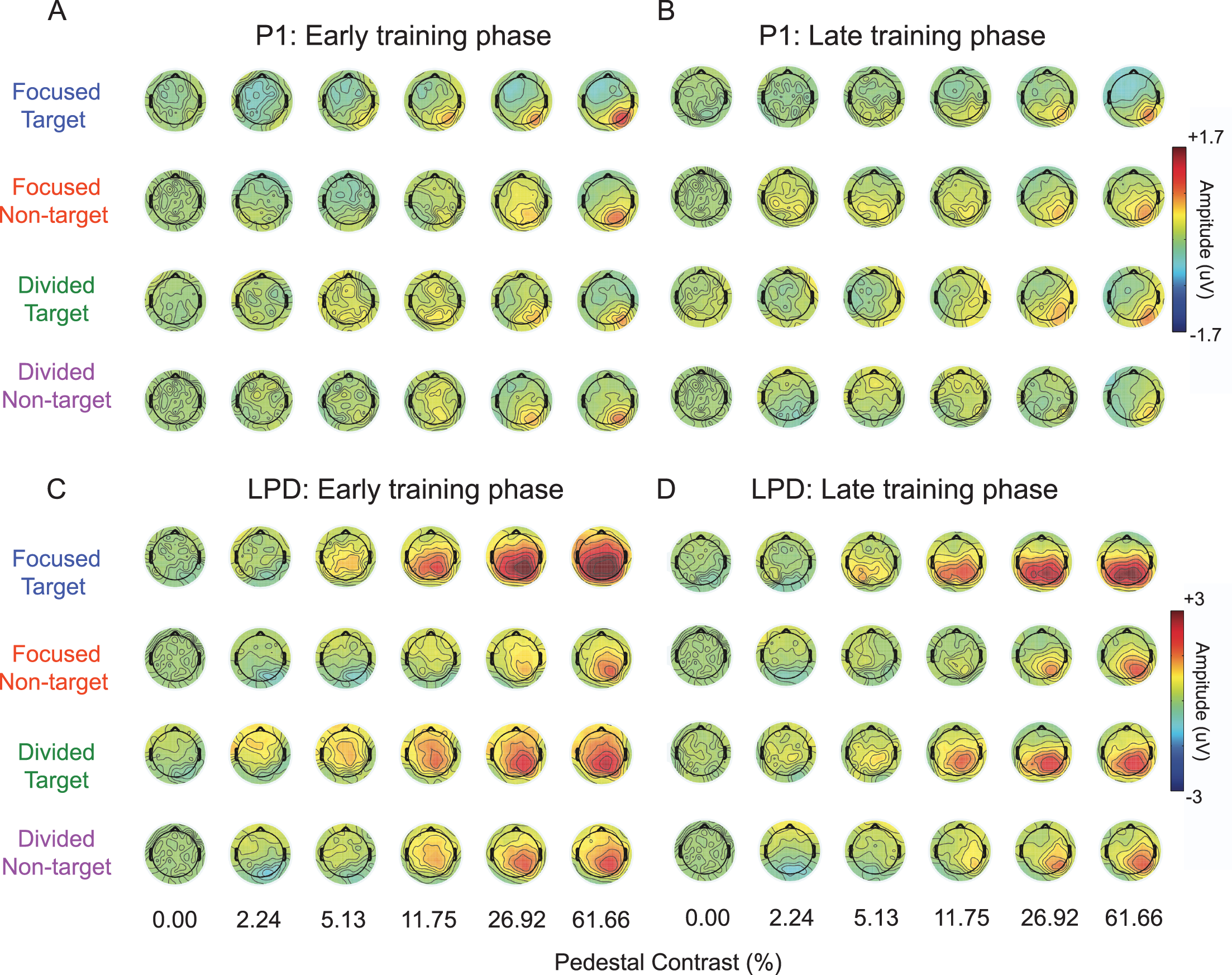

Supplement: S1 Fig — The left and right sides of the head model represent electrodes ipsilateral and contralateral to the stimulus of interest, respectively. (TIF) [file pbio.2001724.s003.tif]
